# Design guidelines for healing gardens in the general hospital

**DOI:** 10.3389/fpubh.2023.1288586

**Published:** 2023-12-01

**Authors:** Quying Wang, Julia Nerantzia Tzortzi

**Affiliations:** Department of Architecture, Built Environment and Construction Engineering, Politecnico di Milano, Milan, Italy

**Keywords:** healing garden, hospital, rehabilitation effect, guidelines, Wuhan

## Abstract

**Introduction:**

Despite being recognized as a cost-effective method to enhance physical and mental health, Healing Gardens remain insufficiently popularized in outdoor spaces of hospitals. This paper aims to introduce a new perspective and offer guidelines for their implementation within general hospitals.

**Methods:**

A methodology is proposed for formulating hospital-specific guidelines, encompassing the extraction of successful Healing Garden features from case studies, definition of key components grounded in theoretical frameworks, validation of essential features through user questionnaires, and comprehensive site analyses.

**Results:**

The methodology was applied in a case study at Zhongnan Hospital in Wuhan. This research presents a novel perspective and robust methodology for implementing Healing Gardens in general hospital settings, potentially improving physical and mental health in a cost-efficient manner.

**Discussion:**

This work aims to encourage the adoption of Healing Gardens as preventive medical tools in more healthcare settings. By providing a comprehensive methodology and a case study illustration, this research endeavors to stimulate broader acceptance and utilization of Healing Gardens in healthcare environments.

## 1 Introduction

The connection between nature and human health has deep historical roots, dating back to 1000 B.C. when civilizations in Egypt and India recognized the healing properties of nature and cultivated medicinal garden (1). In the Middle Ages, cloister gardens in hospitals and monasteries provided patients with nature's calming effects (2). However, the modern medical paradigm, with its emphasis on pharmaceuticals and the advances in technology, has often led to a neglect of the psychological and social health benefits of nature (3, 4). Moreover, the concept of health has evolved to encompass not only physical wellbeing but also mental and social wellbeing (5).

It is crucial to delve deeper into the multifaceted aspects of health. Exposure to green spaces provides a platform for physical activities (6), which are widely acknowledged as a critical protective factor against numerous non-communicable diseases, encompassing cardiovascular diseases, hypertension, diabetes, obesity, mental disorders, and various forms of cancer (7–12). Additionally, urban green spaces offer opportunities for social activities, fostering neighborhood bonds, influencing the perception of safety, and enhancing social cohesion (13–15).

Contemporary research consistently demonstrates that nature has a versatile and positive influence on human health (11). In general, the effects of green spaces on health have several aspects: they reducing exposure to environmental stressors such as air pollution (12), noise (13), and heat (14); building capacities, including encouraging physical activity (16), facilitating social cohesion; restoration capacity, including attention restoration (15) and psychophysiological stress recovery (17); and other benefits, such as reducing transmission of epidemics (18). A well-designed urban green space is closely linked to the “One Health” concept (19), which seeks to promote overall health of the environment, animals and people.

The emergence of biophilic design concepts has encouraged the incorporation of natural elements and processes into the built environment (20). This concept is underpinned by the belief that exposure to natural environments and features has a positive impact on human health and wellbeing (21), a notion well-supported by extensive research (22). These concepts have given rise to the built of theoretical framework for the relationship between nature and the built environment (23). Several theories have been proposed and verified to explain why exposure to nature is beneficial, including Attention Restorative Theory (ART) (22), Stress Recovery Theory (SRT) (24), The Kaplans' Environmental Preference Theory (25) and Roger Ulrich's Supportive Design Theory (SDT) (26). According to ART, nature environments can help restore the targeted attention resources (27–30). SRT proposes that exposure to certain natural environments can alleviate the physical, psychological and behavior damage caused by stressors. Environmental Preference Theory further provides a structure format to assess user behavior and attitudes toward the degree of preference for landscape, incorporating elements such as coherence, legibility, mystery and complexity (31). SDT extends the contents of SRT and explores how a designer can utilize the built environment to reduces stress. Four stress-relieving resources which are important in the healthcare environment were identified: movement and exercise, social support, sense of control, and nature distractions (5, 29).

The application of this theoretical framework holds significance not only in theory but also in built environmental practice (20), particularly within hospital environments (32). An increasing number of hospitals are integrating gardens as the tool to improve health into their development (33, 34), as these gardens possess natural qualities that reduce stress, enhance wellbeing, and enrich the hospital environment (35). It has been proven that patients who have access to nature experience positive change of their mood (4), and tend to have shorter hospital stays, reduced medication requirements (36). They also report improve the ability to exercise, decreased feeling of isolation and alleviated depression (37). In addition to patients, therapeutic hospital gardens (34) also have positive effect on patients' families, staff and visitors (38–41): they provide space for staff (42) and family member of patient (43) to recuperate from stress. Hospital gardens are often more effective than indoor area for respite and relaxation, provide a quiet and peaceful interaction place for patients and visitors (43).

Compared to smaller cities, big cities face a more severe situation regarding the requirement of access to restorative environments (44). The fast-paced life in cities imposes greater demands on information processing and dealing with social stressors, which can have negative impacts on residents' physical and mental health (45). Wuhan, with its 11 million population, and being one of the epicenters of the outbreak in China, had over half of its park accessibility levels not matching their population density (46). With high settlement density (47) and more psychological demands for contact with nature (26), there are more reasons to require restorative gardens, especially after experiencing the epidemic.

The problem of an insufficient hierarchical medical system has existed in China for years, resulting in an “inverted triangle” of health resource allocation phenomenon where primary and secondary care only treat 20% of cases while tertiary hospitals (mostly general hospitals) solve 80%. Due to the public's lack of trust in the abilities of lower-educated practitioners (48), patients tend to go to higher level hospitals rather than primary care when they require medical treatment (49). Meantime, because of the relationship between people in China, when someone falls ill, the patient's family or friends will accompany with them to the hospital. In addition, patients and visitors will be prone to mental fatigue and pressure due to the cold and to the overload of electronic information in the infusion center, surgery waiting area, registration area and other places. These situations thereby aggravate the rapid development of tertiary hospitals (41) and increase the pressure on the medical system and on the patients to an unprecedented level. Studying the design of the healing garden in a general hospital in China may enhance the possibility of relieving the pressure on people's intensive use of the hospital (50) and reducing the risk of virus infection (44).

However, the use of restorative design in general hospitals is not positive, with only a few nursing home, community gardens and individual attached hospitals being designed according to the healing landscape theory (32). The design and practice of healing gardens in general hospitals which focus on human health (includes physical, mental and social health) are very limited and has only begun to emerge in the last 5 years (45).

The authors aim to develop new guidelines for healing gardens in general hospitals based on the theoretical framework of biophilic design, healing gardens, and related research. Specifically, this study focuses on the design of outdoor spaces as restorative gardens and their potential application in the case of Zhongnan Hospital in Wuhan, which was selected for the study. The significance of healing garden design in the general hospital context is emphasized, exploring how it can relieve pressure on the medical system, reduce the risk of virus infection, and promote the physical and mental health of patients, visitors, and hospital staff. Overall, this study seeks to provide a practical and comprehensive framework for healing garden design and implementation in general hospitals, particularly in the case of Zhongnan Hospital, to enhance their social significance and to contribute to the development of cost-effective preventive medical tools (51), potentially leading to the prevalence of healing landscapes.

## 2 Materials and methods

The methodology proposed for defining guidelines for a healing outdoor environment at Zhongnan Hospital is based on three key elements: a theoretical framework, case study, questionnaire, and site analysis.

The theoretical framework is consist of factors that could evaluate healing garden, including Kaplan's four environmental preference factors (25), Ulrich's four stress reduction factors and other factors. Specifically, we consider the environmental preference factors of coherence, legibility, mystery, and complexity, as well as Ulrich's stress reduction factors, which include movement and exercise, social support, a sense of control, and nature distractions (52). In addition to these factors, the assessment of a healing garden also takes into account other important aspects such as the risk of injury, security, and accessibility (34).

The study draws on the success of three healing gardens, namely the Joel Schnaper Memorial Garden, the Elizabeth & Nona Evans Restorative Garden, and the Einstein Medical Center in Philadelphia. These gardens were assessed using Roger Ulrich's supportive design theory and Kaplans' environmental preference theory. The design principles for successful projects are then derived from the analysis of these gardens.

The questionnaire aims to understand how the outdoor environment of the hospital site is being utilized, and to identify priorities and preferences of users (hospital staff, patients, visitors, etc.). The questionnaire comprises both closed and open-ended questions to ensure that users can express their opinions about the healing garden. Given the pandemic situation when delivering the questionnaire survey (in 2019), the questionnaire was distributed online. The results of the questionnaire, including all questions and responses, are presented in the “Results” chapter of this study.

The site analysis, on the other hand, aims to identify the potential and opportunities for using the site. Wuhan Zhongnan Hospital is situated in the central cultural district of Wuhan, adjacent to the Chu River and Han Street, and on the shore of the scenic East Lake. It is a large modern hospital that integrates medical treatment, teaching, scientific research, preventive health care, and community medical services, with a construction area of 90,500 square meters and an outdoor environment of 39,500 square meters (47, 48). To understand the site, various analyses were conducted, including district belonging analysis, land use analysis, existing vegetation, building entrances, architecture function analysis, existing facility, flow analysis, and legibility analysis based on landmarks, nodes, edges, pathways, and the district analysis. Based on these analyses, a SWOT (strengths, opportunities, weaknesses, and threats) analysis was performed to summarize the findings.

By combining the findings from the literature study, the questionnaire, and the site analysis, we can define guidelines for the design of a healing outdoor environment at Zhongnan Hospital. These guidelines will consider the theoretical framework of healing gardens, as well as the specific needs and demands of the hospital's patients, staff, and visitors. Ultimately, the goal of this methodology is to create a practical and comprehensive framework for the design and implementation of healing gardens in general hospitals, with the potential to promote physical, mental, and social health, while also providing cost-effective preventive medical tools.

## 3 Results

### 3.1 Existing healing garden analysis with theoretical framework

In this study, historical and typical healing gardens were researched based on the Kaplan (49), Ulrich factors (52) and others factors as the theoretical framework. Specifically, the study examined the Joel Schnapner Memorial Garden, the Elizabeth & Nona Evans Restorative Garden, and Einstein Medical Center Philadelphia. The basic features and characteristics of these gardens were drawing from an extensive collection of resources, including books, journals, and online materials, and these characteristics are reorganized in Table 1 based on the factors of theoretical framework identify their response to the indicators. Following are the outstanding characteristics of studies healing gardens.

**Table 1:** Existing healing garden analysis data.

**Factors**	**Subfactors**	**The Joel Schnaper Memorial Garden**	**The Elizabeth & Nona Evans Restorative Garden**	**Einstein Medical Center Philadelphia**
Kaplan factors	Coherence	The garden is small but well-organized.	Paths are clear and readable. Different areas such as the meditation garden and the horticultural health garden are clearly separate.	The outdoor environment is organized with lines and curved patterns.
	Legibility	The overall planning is very intuitive, patients can see where they want to go in any place in the garden.	This healing garden is composed of three unique landscape areas, each has its own boundary.	Landmarks and paths help make this garden legible.
	Mystery	There is not much mystery in the gardens as patients can easily see the entire garden at a glance.	The size of the garden creates mystery because visitors can only know it by exploring it entirely. Using walls with openings, and different height of plants could also increase the feeling of mystery.	The garden is relative open, but walls, plants, different areas help to increase its mystery.
	Complexity	The Activity pavilion is flexible, is movable and can be combined as needs be to hold large or small activities.	The garden has many activities for visitors to engage in like contemplation, exploring nature, teaching activities, planting etc.	Different kinds of vegetation, water landscape stimulate the visitor's senses.
Ulrich factors	Movement and exercise	Working with plants, Walking through the garden.	Walking through the gardens, a lot of space to walk in and many activities can be engaged in.	A lot of space and area for visitors to walk in, long path for people to wander.
	Social support	Patients can help each other at project and communication in the demonstration farm.	Spend time with family, friends and staff, gain physical and mental vitality through cultivation activities and communication with other people.	Set seating and rest area and gathering area to enjoy time with others
	Sense of control	Have opportunity to take care of plants and display in the demonstration farm.	Have multiple spaces for different people to choose from, walls provide security, and allow people to visit and exit the garden freely	Have different areas to engage with and choose from
	Natural distractions	Plants offer distraction.	Sounds of nature, vegetation diversity, and the water landscape on the wall, attract people and bring people different sensory experiences, different activities in the garden for visitors.	Sounds of water from the wall with water elements, diversity of vegetation
Other factors	Risk of injury	Flat ground, use plants as barrier rather than sharp concrete wall.	The sidewalk has a small slope which can minimize fatigue and increase the safety of people walk on it	The slope of the ground has been minimized, planters and other elements are rounded rather than having sharp corners.
	Security	It's surrounded by walls on all sides.	It's surrounded by plants and walls, and it's been built in a botanical garden.	It has open and half open space, relatively quiet and safe.
	Accessibility	The space is designed to make wheelchair use easy	This is a place where everyone can enjoy, while the privacy and publicness of the garden are in balance.	This area is relatively open, it's in front of the hospital, beside the parking area and connected to the courtyard of the hospital.
Overall statement		The overall planning is very intuitive. Patients can see where they want to go in any place in the garden. At the same time, the design of the garden is very flexible, not only because of the flat roads and the accessibility of the area, but also the movable pavilion can be changed according to the needs of patients. For example, the tent can be moved and combined as needed to hold large or small activities. It can also be used as a shady place for patients to communicate with each other.	In general, the overall layout is reasonable and human, emphasizing and focusing on natural landscapes, having multiple spaces for different people to choose from, and emphasizing the physical and psychological treatment of people at the same time. The sensory garden is easy to experience with the five senses.	The designer set wandering area, gather area, rest and seating area, plants area for people to choose to have their rehabilitation training. The sound made from the water landscape wall attracts people and attenuates the surrounding noise and increases the sense of interest with a diversity of local vegetation, straight and curved facilities.

The Joel Schnaper Memorial Garden, built in 1995 on the rooftop of the Terence Cardinal Cooke Health Care Center in New York, serves as a example of a healing functional roof garden in the early stages of this concept's development. Designed to provide a healing space for the older adult and individuals with developmental disabilities and chronic illnesses, this garden has received national and regional design awards, including the prestigious 1995 ASLA Merit Award in General Design. It underscores the notion that well-planned and well-operated gardens can effectively reduce stress and promote a sense of wellbeing among long-term healthcare patients (53–55).

The garden's design places a strong emphasis on addressing the specific needs of individuals with HIV, encompassing considerations like physical strength, sensory abilities, sunlight sensitivity, orientation, and the importance of physical activity, social interaction, privacy, and independence. Furthermore, the garden hosts a horticultural therapy program integrated with the center's physical and occupational therapy initiatives. Temporary kiosks have been strategically employed to cater to diverse needs during different events and times, while a demonstration farm complements patient communication, exercise opportunities, and fosters a sense of accomplishment. The garden layout is intuitively planned, facilitating easy navigation, and the use of plants both as privacy shields and zoning elements enhances the overall experience.

The Elizabeth & Nona Evans Restorative Garden built in 2005 in Cleveland Botanical Garden in Cleveland, stands as another noteworthy healing garden. Unlike the Joel Schnaper Memorial Garden, it is open to the general public, rather than being limited to patients, individuals with disabilities, or staff members. This garden also received recognition, winning the ASLA 2006 Professional Awards. It embodies the concept of a healing garden designed to cater to a broad spectrum of human conditions (56–58).

The design of the Elizabeth & Nona Evans Restorative Garden is distinguished by its division into three primary areas, each serving different purposes: spaces for contemplation, exploration and learning, and areas designated for horticultural therapy. The landscape design emphasizes natural elements and incorporates various materials and features such as waterfalls and flowing water to provide diverse sensory experiences. Different planting areas cater to individuals with different needs, incorporating plants with varying flowering periods to extend the garden's aesthetic appeal throughout the year. The garden's attention to detail is evident through features like handrails and braille on railings, making it accessible and inclusive.

The Healing Garden of Einstein Medical Center, built in 2013 in East Norriton, exemplifies the concept of a “landscape for healing” and serves as a compelling case study for the outdoor environments of large hospitals. The garden's design includes various functional areas, such as walking paths, leisure seating spaces, gathering areas, distinctive waterscapes, and dedicated plant areas, all of which contribute to providing rehabilitation and therapeutic venues for patients. Moreover, the inclusion of walls and sloped terrain within the garden creates a sense of enclosure, contributing to the overall healing experience. The garden uses water features to mitigate noise and has carefully chosen a diverse range of plant species suitable for the local climate, promoting an environment conducive to healing (59–61).

The characteristics of the studied healing gardens discussed above will be systematically reorganized in Table 1 according to a theoretical framework. This approach aims to enhance our comprehension of how these factors manifest in real-world projects. By aligning these features with the structure of the chosen theoretical framework, we seek to gain deeper insights into their practical applications and performance in healing garden design.

### 3.2 Questionnaire

To understand how the outdoor environment of Zhongnan Hospital is being used and what activities are important to users, an online questionnaire survey was released on April 3, 2020. The questionnaire consisted of closed and open-ended questions to capture users' opinions about the garden. Due to the COVID-19 pandemic, the questionnaire was conducted online. As of June 8, a total of 418 valid responses were collected. These responses were analyzed to identify the most important features and activities for users and to inform the development of guidelines for the hospital's outdoor environment.

#### 3.2.1 Structure of the questionnaire

The questionnaire survey was conducted to collect basic information about the usage of the outdoor environment at Zhongnan Hospital. It aimed to gather information on the basic characteristics of respondents, their activities in the existing outdoor environment, and the duration of their visits. The questionnaire also aimed to elicit respondents' evaluations of the site from several aspects, including their expectations, satisfaction, and dissatisfaction with the site, as well as their demands for staff gardens and outdoor rehabilitation projects.

The questionnaire included both closed and open questions to ensure that all features of the outdoor environment were considered. A detailed version of the questionnaire can be found in Appendix 1.

#### 3.2.2 Data analysis

**Figure 1:**
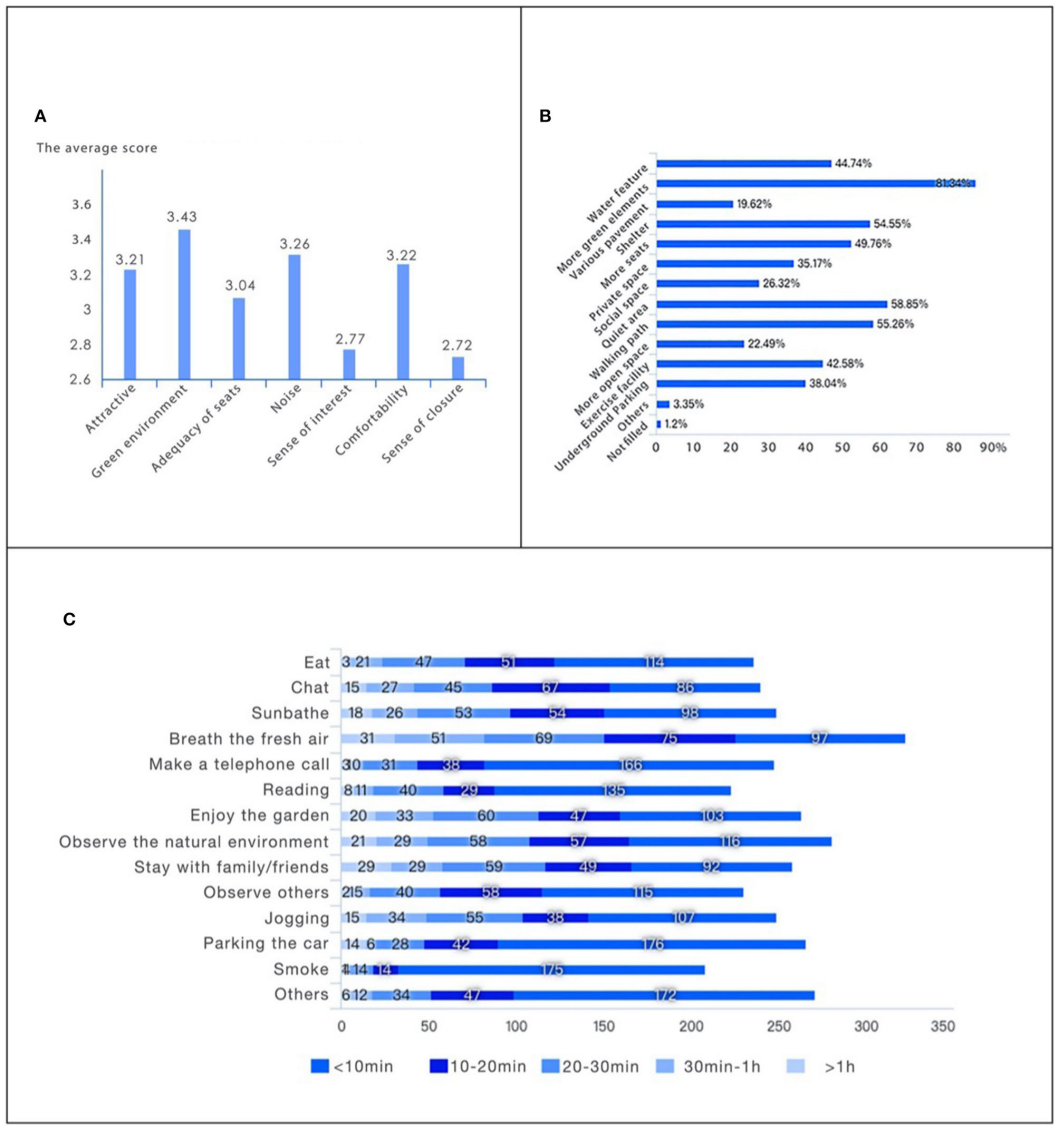
Questionnaire results. **(A)** From 1 to 5, how do you rate these aspects of the outdoor environment? **(B)** What features do you expect in the existing outdoor environment in the Zhongnan Hospital? **(C)** What do you generally do in the existing outdoor environment? How long does it take?

Based on the analysis of Table 2 and Figure 1 (the full and detailed results from questionnaires are presented in Appendices 2, 3), which reflect the responses to the questionnaire, the most important features desired by users for the outdoor environment of Zhongnan Hospital are as follows:

**Table 2:** Questionnaire analysis.

**Questions**	**Responses**
Your identity is?	Hospital staff(239) Visitor(66) Patients(5) Others (107)
Your age is:	< 20(81) 20–30(184) 30–40(82) 40–60(61) >60 (9)
Are you satisfied with the existing outdoor environment?	Very satisfied(15) Satisfied(49) Neutral(152) Dissatisfied(136) Very dissatisfied (63)
At what time of the day do you usually use the outdoor environment?	Morning(103) Noon(39) Afternoon(117) Night (150)
How important is it for you to have a garden outside Zhongnan Hospital?	Not important(10) Not so important(17) Neutral(65) Important(177) Very important (147)
What do you generally do in the existing outdoor environment? How long does it take?	Parking the car < 10 min(176) Smoking < 10 min(175) Make a phone call < 10 min(166) Reading < 10 min(135) Observe the nature environment < 10 min(116) Eat < 10 min(114) Breath the fresh air 10–20 min(75) 20–30 min(69) 30 min−1 h(51) > 1 h(31) Chat 10–20 min(67) Stay with family/friends 20–30 min(59) >1 h(29) Jogging 30 min−1 h (34)
Do you want some rehabilitation projects to be carried out outdoors?	Yes(329) No (86)
From 1to 5, How do you rate these aspects of the outdoor environment?	Attractive (3.21) Green environment (3.43) Adequacy of seats (3.04) Noise (3.26) Sense of interest (2.77) Sense of comfort (3.22) Sense of enclosure (2.72)
Do you want a place to seat? (alone/with one or two people/in groups)	Alone 140 With one or two people 246 In groups 26
If you are a hospital staff in Zhongnan Hospital, do you need a dedicated outdoor garden for you and your colleagues?	Yes(365) No (44)
What features do you expect in the existing outdoor environment in the Zhongnan Hospital?	More flower and green elements(340) Quiet area(246) Walking path(231) Shelter(228) More seats(208) Water feature(187) Exercise facility(178) Underground parking (159)
If you are a healthcare provider, what is the stress index (from 1 to 5) of your relationship with patients?	1(31) 2(52) 3(177) 4(77) 5 (41)
What are your satisfactions and dissatisfaction with the existing outdoor environment of Zhongnan Hospital?	Too many cars, not enough parking space, need underground parking(68) No segregated pedestrian, car and trolley-bed roads(18) No shelter and flat road for transferring patients(13) Lack of convenience store, exercise facility, public seating, green elements (3)

• *More parking space, preferably underground*.

According to the results of the question 11 in the questionnaire, 38.04% of respondents indicated that they expect the hospital to have underground parking. This expectation was also evident in the responses to the open question 13: “What are your satisfactions and dissatisfactions with the existing outdoor environment of Zhongnan Hospital?” where many users expressed dissatisfaction with the number of cars in the area and suggested the need for more parking. For instance, from the answer sheet 6 “There are too many cars, it is very unfriendly to pedestrians, and the outdoor environment is not well utilized! Basically, it's only for a parking function.” Sheet number 13 “There are too many cars, which affects the traffic and is not safe, and affects the air quality. It is better to introduce the parking lot underground.” The need for underground parking was mentioned in various answer sheets, such as sheets 3, 17, 19, 20, 28, 29, 32, 33, 41, 49, etc. In summary, according to the questionnaire, the outdoor space is mainly used for parking functions. It is noisy, unsafe, has insufficient parking spaces, and parking spaces are occupied by daily traffic. Users hope to have an underground parking lot.

• *Rapid medical access, with shelter and flat roads, for hospital staff to transfer patients*.

This important feature was also mentioned in the open question, for example in sheet number 189: “The road from the outpatient department of our hospital to Building 6/7 is narrow and bumpy. There is no segregation between pedestrians, vehicles, and hospital gurneys. No widening, not much change.” Sheet 223 also commented, “Repair the road. It's really hard work to turn a patient around. I'm so tired and sweaty, and the distance between buildings is still far away. I really need the road to be repaired.” Similar comments were made in other answer sheets (240, 250, 255, 263, 275, and 276). Without rapid medical access, it is not only hard for doctors to transfer patients but also very dangerous for patients. This issue was also mentioned in sheets 255, 275, and 263.

•*A divided path for medical, walking, and vehicular traffic*.

A divided (segregated) path system was also mentioned as an important feature, as it can contribute to better traffic flow and pedestrian safety in the hospital. This feature was suggested also in response to the need for rapid medical access.

• *Rehabilitation projects carried out outdoors*.

According to question 7 of the questionnaire, the majority of respondents (78.71%) hoped for the inclusion of outdoor rehabilitation projects in the hospital's outdoor environment.

• *More flowers, green elements, and water features*.

Question 11 of the questionnaire reflects users' expectations for the transformation of Zhongnan Hospital's outdoor environment in the future. The most popular response was the desire for more flowers and greenery (81.34%), followed by water features (44.74%).

• *Quiet areas and more paths for walking*.

Answers to question 11 showed that users desire quiet areas and more paths for walking (58.85%), as well as more walking areas (55.26%). Walking places were mentioned frequently in the open question, with comments such as “There are basically no pedestrian passages, and there is basically no reasonable planning in the hospital, which is extremely chaotic” (sheet N51).

• *More basic facilities, such as shelters, seating areas, and exercise facilities*.

The survey data from question 11 showed that shelter (54.55%) and more seating options (49.76%) were important elements that users desired. In addition, for those who want to have an environmental space where they can sit down, the majority of respondents (58.85%) preferred a seating area where two or three people can sit together. A significant number of respondents (33.49%) wanted a place where they could sit alone, while 6.22% preferred seating in groups. Therefore, a variety of seating arrangements could be an important element in this outdoor space.

• *Staff garden for stress relief*.

Medical staff and personnel are under a considerable amount of pressure in their work (from 1 to 5, average 3 points in the reply to question 12), and they require a space where they can relax and release their stress. The survey data showed that the majority of respondents (87.32%) wished to have an outdoor garden that could be used exclusively by medical staff and personnel (question 10).

• *Convenience stores or places to purchase food and other items*.

The need for a convenience store or someplace to buy food and other essentials was identified as an essential requirement in the survey. In one of the survey responses (Number 245), the respondent wrote: “There are too few places to buy things, which can't meet the needs of patients and medical staff. Especially in terms of diet, there is no way to provide a variety of diets. There are no small shops, which may be related to the geographical location and are relatively close.” Another respondent (Number 236) expressed the desire to have a coffee shop.

• *Outdoor environment with a sense of interest, enclosure, and tranquility*.

The evaluation of the existing outdoor environment (question 8) revealed that the lowest score was given to the sense of interest of the outdoor environment. The external environment of the Zhongnan Hospital was mainly occupied by parking, making it hard to find any sense of fun. The score for the sense of enclosure was also low, with a score ranging from 1 to 5. The score for the sense of enclosure was 2.72, indicating that there are few places that give people a sense of enclosure in the external environment of the hospital. The score for noise was relatively high (3.26), indicating that a quiet environment is demanded by users.

In summary, the survey results showed that the outdoor space required by users should include basic facilities such as shelter and various seating options, a staff garden to help staff relieve stress, convenience stores and food options, and an outdoor environment with a sense of interest, enclosure, and quietness. These findings provide valuable insights into the features and requirements of outdoor spaces in healthcare settings.

### 3.3 Site analysis

**Figure 2:**
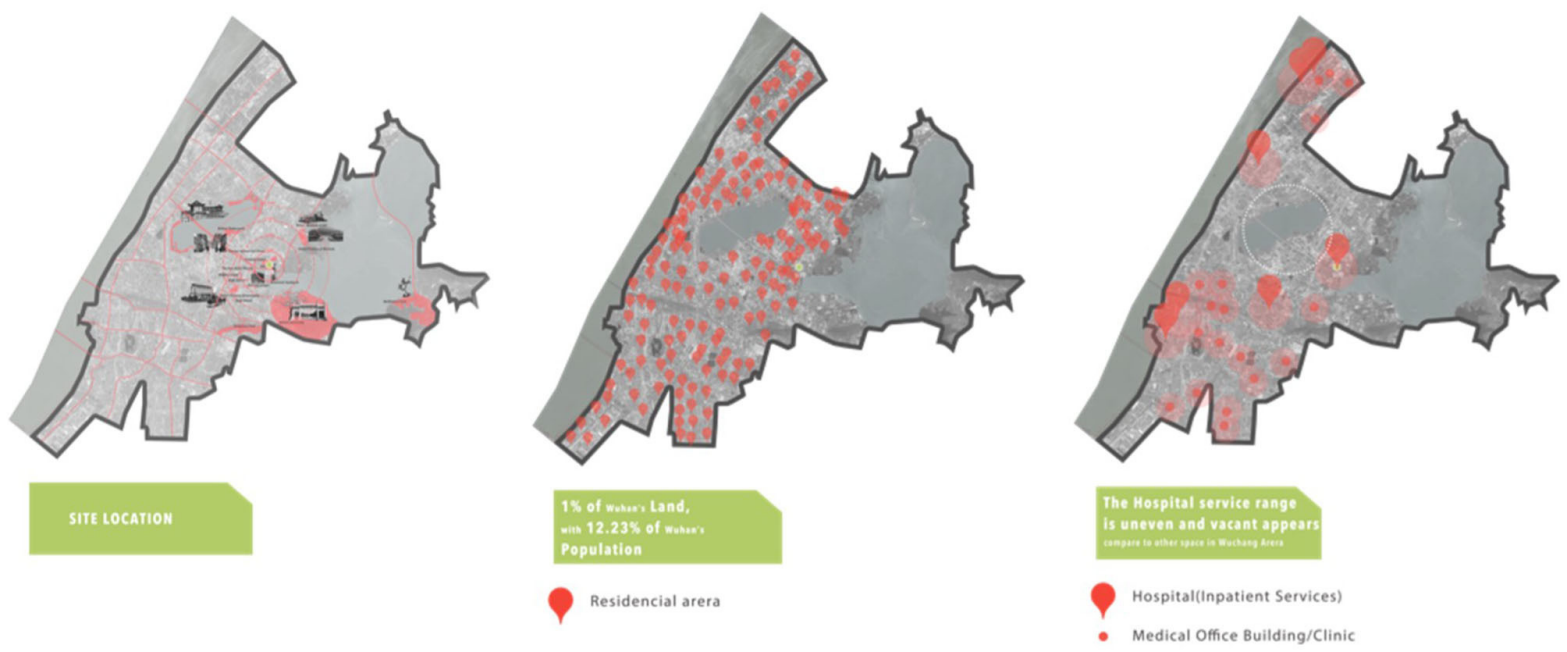
District analysis.

Zhongnan Hospital is located in Wuchang District, Wuhan City, Hubei Province, China. According to a study conducted in the Wuchang District, it was found that 12.23% of Wuhan residents lived in Wuchang, which comprises only 1% of Wuhan's total area (62, 63). Although the green area in Wuchang is not insignificant, there are only a few public parks and gardens available for public use. By mapping the major hospitals and clinics in Wuchang and the services they offer, it was discovered that the area lacked hospital services, with only one 3A hospital, Zhongnan Hospital, operating in the region (Figure 2).

The land use analysis revealed that most of the functions in the area were residential, followed by education (a Wuhan University campus and a primary school are located nearby). There is a large vacant area in the region, primarily used for garbage and car parking, with only a few recreational areas, and a lack of public green spaces. The site has four main medical buildings, including an internal medicine complex, a women and children's center linked with an orthopedic center, an emergency center, and a department of oncology.

According to the results from the site survey, it's easier to understand issues exposed from the questionnaire: almost 90% of the outdoor area was used for parking, with only a few green elements such as trees besides main roads and small green area with pavilions. Despite most of the area being used for parking, there is still a shortage of parking spots, and usually people need more than 10 min to find a parking space. This lack of parking space has created a messy division between cars and pedestrians, with patients on sickbeds having to navigate around cars, posing a safety risk. Additionally, the site lacks facilities, with only two out of nine pavilions being used and with only a few seats in front of the main medical building. During the observation, people were seen sitting on the edges of flower ponds while eating lunch or making phone calls.

**Figure 3:**
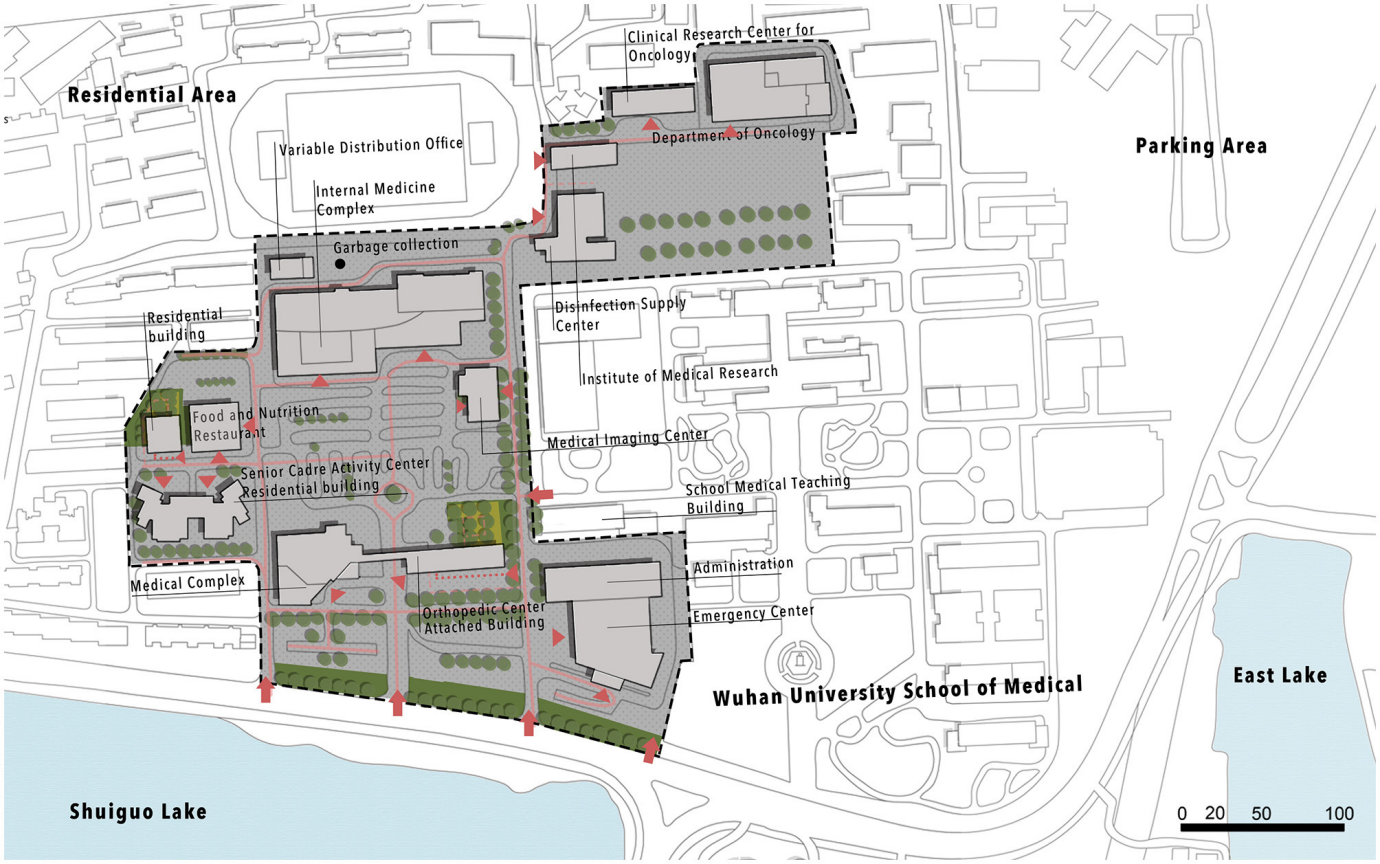
SWOT analysis.

From Figure 3 and Table 3, the SWOT analyses revealed the strengths, opportunities, weaknesses, and threats of the site. The strengths included its good location and connection with surrounding areas, a large space with a lake in front of the site, existing trees, most clients having a view of the garden, and historical value. Opportunities included the large area available for redevelopment, the opportunity for creating a healing environment, shelters and seating areas, underground parking, and a space for outdoor therapy. Weaknesses included abandoned parking areas, a lack of shelters and seats in the garden, no underground parking areas, no private areas for individual and family visits, a physical barrier created by the driveway dividing the site, a lack of seating and recreational features. Threats included traffic noise, high maintenance costs, persistence of disconnection in the site, and insufficient parking.

**Table 3:** SWOT analysis.

**Direction**	**Content**
Strengths	Good location and good connection with surrounding areas A lot of existing trees Large space There is a lake in front of the Site. Most clients have a view on the garden. Historical Value
Opportunities	Large area that could be re-designed to Create a more healing environment. Creating shelters and seating areas, Creating underground parking areas Space for bringing therapy outside. Large space for installing the various facilities.
Weaknesses	Abandoned parking areas Lack of shelters and seats in the garden Lack of private areas for individual and family visits The driveway creates a physical barrier, dividing the site into parts Lack of seating and recreational features No pathways Only two main functions in the outdoor spaces
Threats	Traffic noise High maintenance costs Parking area is not big enough

These results were obtained by conducting a site visit, mapping the existing site functions (using AutoCAD and Photoshop), vegetation distribution, architecture function map, entrances distribution, existing facility utilization, and Lynch analysis (64) to analyze the key physical elements, including districts, landmarks, nodes, edges, and pathways.

In conclusion, the study provides an insight into the current state of the Zhongnan Hospital site in Wuchang District, identifying its strengths, weaknesses, opportunities, and threats. The findings suggest that improvements are necessary to make the site more accessible and functional, such as creating underground parking areas, adding more green spaces and recreational features, and providing private areas for individual and family visits. Such changes will help to create a more healing environment and improve the overall patient experience.

### 3.4 Guidelines for the design of a healing garden for the rehabilitation in Zhongnan Hospital

Based on the findings presented in Table 4, the design guidelines for the healing garden at Zhongnan Hospital were developed. The guidelines consist of three parts: guidelines derived from theory and case studies, guidelines derived from questionnaire results, and guidelines derived from site survey and analysis. Part 1 draws from Kaplan and Ulrich's factors, namely coherence, legibility, mystery, complexity, movement and exercise, social support, sense of control, and nature distractions, as presented in Table 1. However, factors such as risk of injury, security, and accessibility were not considered due to the predominantly flat terrain at Zhongnan Hospital.

**Table 4:** Guidelines for the design of a healing garden for the rehabilitation in Zhongnan hospital.

**Extracted area**	**Guidelines**
Guidelines coming from the case study and the theoretical framework	• Coherence: readable paths, clear edges of different areas, organized well with unified elements (e.g., lines and curved patterns) • Legible: landmarks, clear paths, boundary • Mystery: can help to increase the sense of interest in the site, with the arrangement of the walls and openings helping to create mystery. • Complexity: different green or blue elements, activities to choose from, flexible design. • movEment and exercise: designed walking paths, activities that can be engaged in, horticultural garden or the chance for patients to grow plants. • Social support: private space, seating area that encourage social gathering. • Sense of control: by giving more options to the user such as having multiple spaces or activities for users—or giving opportunities for users to take care of plants—can increase this feeling of control. • Nature distraction: diversity of vegetation and water landscape, sounds of nature (e.g., sound from water, sound from birds) • Reduce the risk of injury, provide place with feeling of security, and consider accessible design.
Guidelines coming from the questionnaire	• More parking places, underground car park • Rapid medical access with shelter and level ground • Segregated paths for different users • Outdoor rehabilitation projects • More green elements • Water features • Quiet area • Walking path • Basic facilities • Staff garden • Convenience store • Sense of interest • Sense of enclosure
Guidelines coming from site survey and site analysis	• Make use of existing strength of the site (good connection with surrounding area, with lots of existing trees, large space, a lake in front of the site, most of clients have a view to the outside) • Take the opportunities in Zhongnan hospital (large area and large underground parking area could be designed, have space to bring therapy outside, large space for installing the various facilities, opportunity to make use of rainwater and the lake in front of site) • Pay attention to weaknesses of site (abandoned parking area, lack of basic facilities such as shelter and seating, needs for private area, recreation features, driveways create a physical barrier, dividing the site into several parts, not enough walking pathways, outdoor space of the site has only parking function) • Take the threats of Zhongnan hospital seriously (traffic noise, high maintenance costs, not enough parking space).

The second part is a summary of the most important features identified from the questionnaire results, as outlined in Section 3.2.2. Part 3 comprises guidelines based on the SWOT analysis of the site, as presented in Section 3.3.

## 4 Design

**Figure 4:**
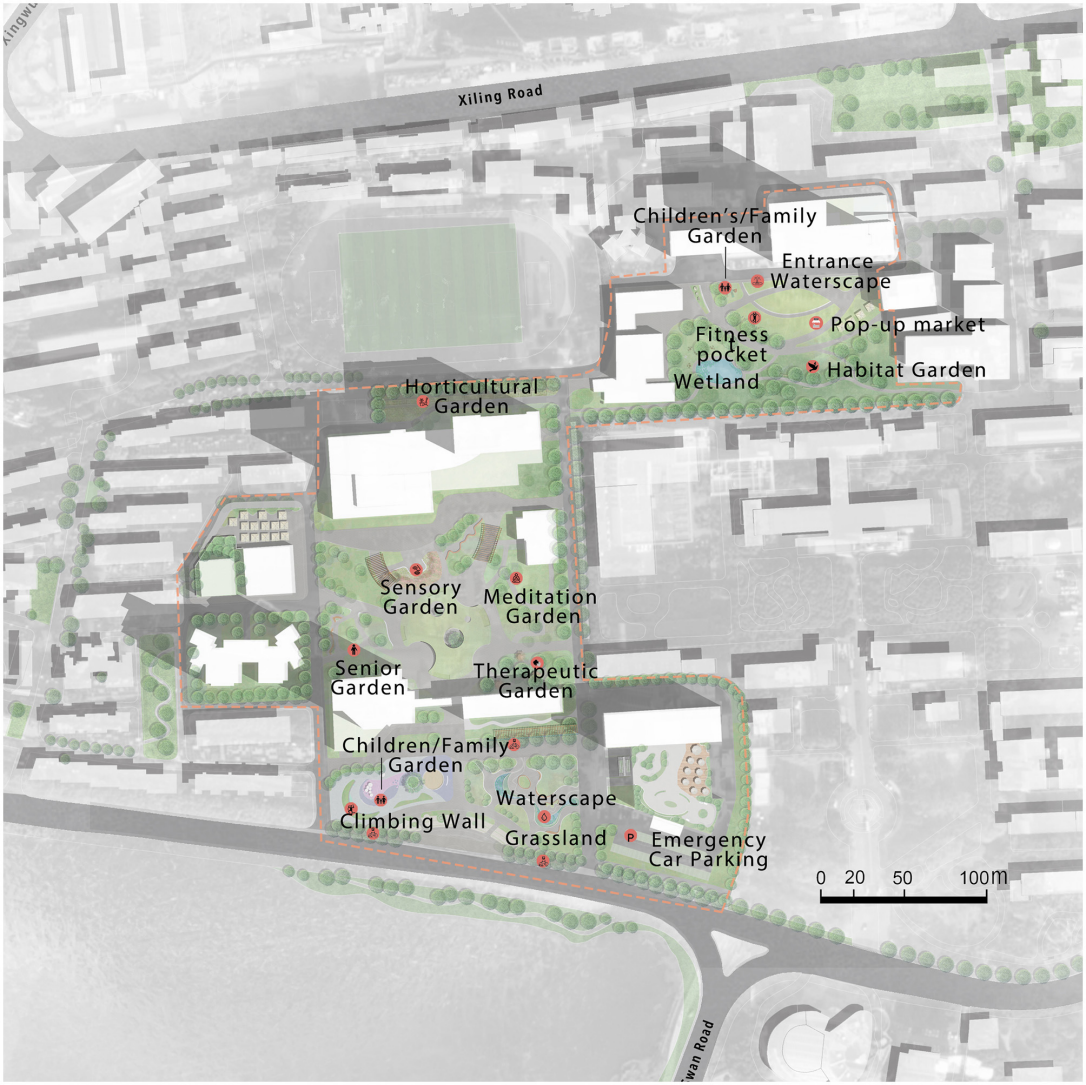
Designed masterplan.

Figure 4 presents the new plan for Zhongnan Hospital, which aims to preserve all existing trees while incorporating more green elements, such as additional trees and various types of vegetation. The plan includes the construction of an underground parking area and an increase in basic facilities, such as more seating, shelters, and shops. Various types of seating arrangements, including areas for individuals to sit alone, areas for two or three people to sit together, and seats for disabled people, are also proposed. The plan proposes different types of spaces, including open space, semi-open space, and enclosed space, to cater to different user demands. In addition, rapid medical access is designed for the transfer of patients. A half-elevated plaza is situated in the center to provide shelter while separating pedestrian traffic. The plan also integrates an area for people to sit and rest, while separating pedestrian traffic from vehicular traffic. Several types of gardens are proposed to cater to different users, including the senior garden for older adults, a children's or family garden in front of the women and children's center, a community garden for surrounding residents, a habitat garden in a relatively private space with many existing trees, and a staff garden on the roof of the medical building. Other types of gardens are proposed based on horticultural therapy theory. They include a sensory garden (to stimulate users' senses); and, rambling, leisure, therapeutic, meditation, and horticultural gardens.

The plan incorporates pop-up markets that cater to various needs, such as movable kiosks for snacks, food, and drinks, places to showcase vegetation planted by patients, and medical camps during epidemic outbreaks. Plants are used as an invisible wall to separate the residential area from the medical area, while also reducing noise from the road. The existing canteen for staff and patients is reconfigured to accommodate separate areas for patients, staff, and other visitors. The plan includes a water-pipe system that connects green roofs, manmade pools, and storage tanks to the east lake, utilizing rainfall effectively.

## 5 Discussion and conclusion

This paper provides a guideline that can be utilized to guide the construction of healing landscapes in general hospitals and use Zhongnan Yiyuan as main example. Research has shown that general hospitals have played a significant role in and after the COVID-19 pandemic, yet their outdoor spaces are often overlooked and mainly used for parking or decorative purposes. Therefore, the proposed healing landscape design guidance program in general hospitals can provide a low-cost and efficient way to establish healing spaces that can enhance people's physical and mental wellbeing during and after the epidemic. The guideline presented in this paper adheres to the principles of healing landscapes and has been developed based on the findings of various investigations, case studies, and research studies.

In this paper, we have examined the characteristics of healing gardens in general hospitals. While many studies have focused on the design of healing gardens for special groups such as the older adults, children, and patients with dementia, few have focused on the needs of general hospital users. Our review of the theoretical framework suggests that restorative environments can effectively promote the restoration of physical, mental, and behavioral health, as well as reduce stress and fatigue.

To design a healing garden, we suggest using Kaplan's four environmental preference factors (coherence, legibility, mystery, and complexity) and Ulrich's four stress reduction factors (movement and exercise, social support, sense of control, and nature distractions) as important indicators. Our review of existing research on healing gardens has provided insights into the key features of a healing garden that correspond to these indicators.

Our survey of users at Zhongnan Hospital revealed the demands of general hospital users for outdoor spaces that are functional and engaging. We also conducted a site survey and analysis that identified strengths, weaknesses, opportunities, and threats related to the design of healing gardens in general hospitals. Our findings suggest that a general hospital such as Zhongnan Hospital could serve not only as a healing garden for its users but also as a therapeutic garden for the surrounding community.

While our study provides valuable guidance for healing landscapes in general hospitals, it's essential to note its limitations. Our focus on Zhongnan Hospital means that the applicability of our findings to hospitals in different geographic and cultural contexts may require customization. Healing garden designs can vary widely due to local factors, such as plant species, climate, and cultural preferences. Thus, our framework offers a general guideline, but specific adaptations may be needed for other locations.

Furthermore, healing gardens in general hospital have the potential to serve as a preventive medical tool during epidemics by providing a safe outdoor environment for various users. Our guidelines for designing healing gardens in general hospitals can be further refined in the future to accelerate the process of universalizing healing gardens and set up a flexible project for various scenarios.

In conclusion, the development of healing gardens in general hospitals can offers cost-effective and efficient solution for enhancing the physical and mental wellbeing of individuals during and after epidemics. By adhering to the guidelines presented in this paper, we aim to inspire more hospitals and communities to integrate healing gardens into their urban landscapes, promoting overall wellbeing in an accessible and sustainable manner.

## Data availability statement

The original contributions presented in the study are included in the article/Supplementary material, further inquiries can be directed to the corresponding authors.

## Author contributions

QW: Writing—original draft. JT: Writing—review & editing.
