# Supplementary material for: Design guidelines for healing gardens in the general hospital
Source: Front Public Health. 2023 Dec 1;11:1288586. doi: 10.3389/fpubh.2023.1288586 (PMC10722422; doi:10.3389/fpubh.2023.1288586)
Supplement: Supplementary file 1 [file Data_Sheet_1.docx]

Supplementary Material

Design Guidelines for Healing Gardens in the General Hospital

Quying Wang, Julia Nerantzia Tzortzi *

*** Correspondence:** Corresponding Author: [Julia.georgi@polimi.it](mailto:Julia.georgi@polimi.it)

# Appendices

**Appendix 1: Questionnaire**

***Investigation on the use of the outdoor environment in Zhongnan Hospital***

1. Your identity is？ [Single choice question]

| ○Patients |
| --- |
| ○Hospital Staff |
| ○Residents Nearby |
| ○Others |

2. Your age is： [Single choice question]

| ○<20 |
| --- |
| ○20-30 |
| ○30-40 |
| ○40-60 |
| ○>60 |

3. Are you satisfied with the existing outdoor environment? [Single choice question]

| ○Very satisfied |
| --- |
| ○Satisfied |
| ○Neutral |
| ○Dissatisfied |
| ○Very dissatisfied |

4. At what time of the day do you usually use the outdoor environment? [Single choice question]

| ○Morning |
| --- |
| ○Noon |
| ○Afternoon |
| ○Night |

5. How important is it for you to have a garden outside Zhongnan Hospital? [Single choice question]

| ○Not important |
| --- |
| ○Not so important |
| ○Neutral |
| ○Important |
| ○Very important |

6. What do you generally do in the existing outdoor environment? How long does it take?

|  | <10 min | 10-20min | 20-30min | 30min-1h | >1h |
| --- | --- | --- | --- | --- | --- |
| Eat | ○ | ○ | ○ | ○ | ○ |
| Chat | ○ | ○ | ○ | ○ | ○ |
| Sunbathe | ○ | ○ | ○ | ○ | ○ |
| Breathe the fresh air | ○ | ○ | ○ | ○ | ○ |
| Make a phone call | ○ | ○ | ○ | ○ | ○ |
| Reading | ○ | ○ | ○ | ○ | ○ |
| Enjoy the garden | ○ | ○ | ○ | ○ | ○ |
| Observe the natural environment | ○ | ○ | ○ | ○ | ○ |
| Stay with family/friends | ○ | ○ | ○ | ○ | ○ |
| Observe others | ○ | ○ | ○ | ○ | ○ |
| Jogging | ○ | ○ | ○ | ○ | ○ |
| Parking the car | ○ | ○ | ○ | ○ | ○ |
| Smoking | ○ | ○ | ○ | ○ | ○ |
| Other | ○ | ○ | ○ | ○ | ○ |

7. Do you want some rehabilitation projects to be carried out outdoors？[Single choice question]

| ○Yes |
| --- |
| ○No |

8. From 1to 5, How do you rate these aspects of the outdoor environment?

|  | 1 | 2 | 3 | 4 | 5 |
| --- | --- | --- | --- | --- | --- |
| Attractive | ○ | ○ | ○ | ○ | ○ |
| Green environment | ○ | ○ | ○ | ○ | ○ |
| Adequacy of seats | ○ | ○ | ○ | ○ | ○ |
| Noise | ○ | ○ | ○ | ○ | ○ |
| Sense of interest | ○ | ○ | ○ | ○ | ○ |
| Sense of comfort | ○ | ○ | ○ | ○ | ○ |
| Sense of enclosure | ○ | ○ | ○ | ○ | ○ |

9. Do you want a place to sit？ [Single choice question]

| ○Alone |
| --- |
| ○With one or two people |
| ○In groups |

10.If you are hospital staff in Zhongnan Hospital, do you need a dedicated outdoor garden for you and your colleagues? [Single choice question]

| ○Yes |
| --- |
| ○No |

11. What features do you expect in the existing outdoor environment in the Zhongnan Hospital? [Multiple choice questions]

| □Water feature |
| --- |
| □More flower and green elements |
| □Various paving |
| □Shelter |
| □More seats |
| □Private space |
| □Social space |
| □Quiet area |
| □Walking paths |
| □More open space |
| □Exercise facility |
| □Underground parking |
| □Other |

12. If you are a healthcare provider, what is the stress index of your relationship with patients? (The pressure increases from 1 to 5) [Single choice question]

| ○1 |
| --- |
| ○2 |
| ○3 |
| ○4 |
| ○5 |

13. What are your satisfactions and dissatisfactions with the existing outdoor environment of Zhongnan Hospital?

_________________________________

**Appendix 2: Questionnaire results**

1. Your identity is？ [Single choice question]


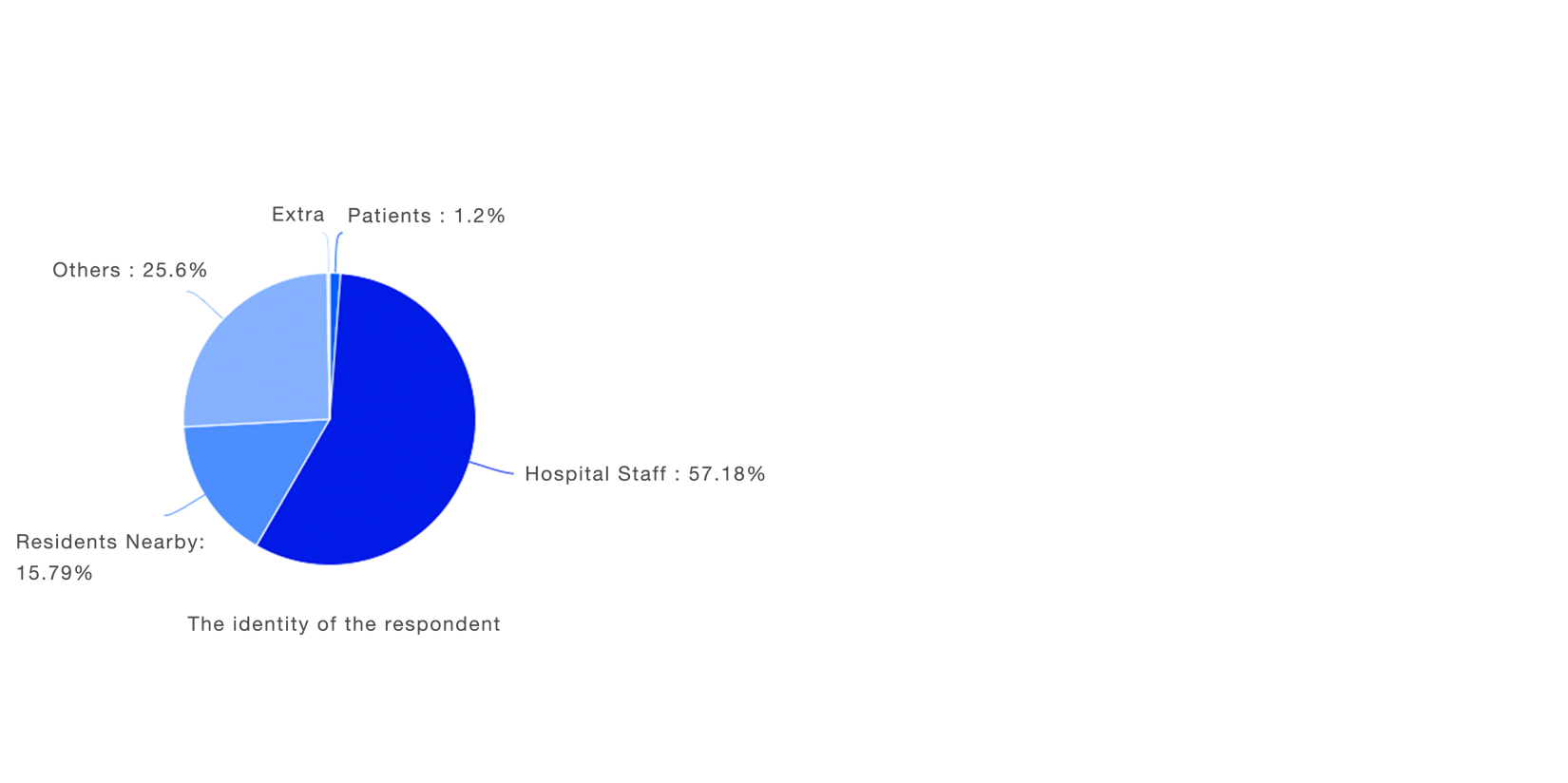


2. Your age is： [Single choice question]


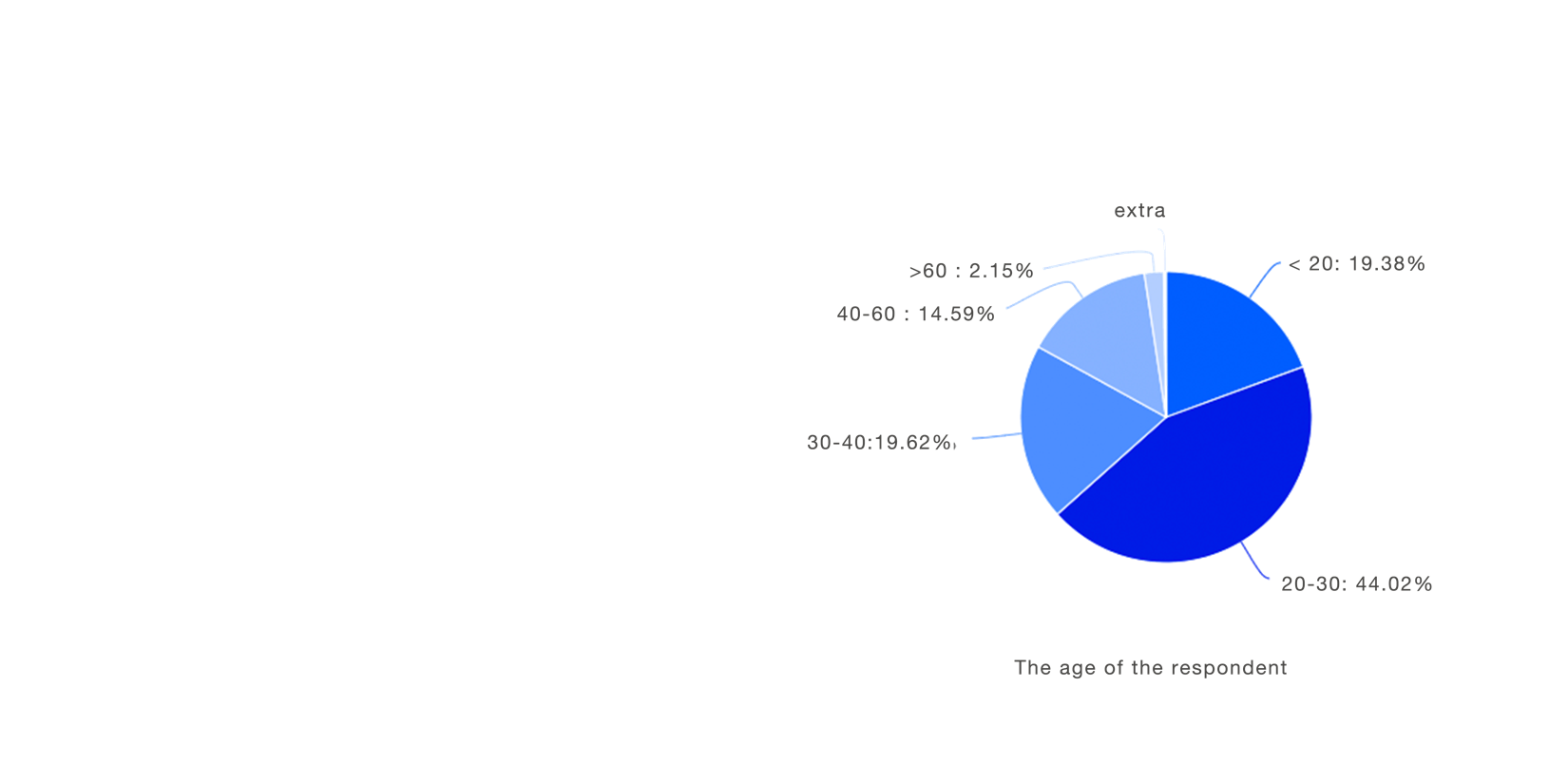


3. Are you satisfied with the existing outdoor environment? [Single choice question]


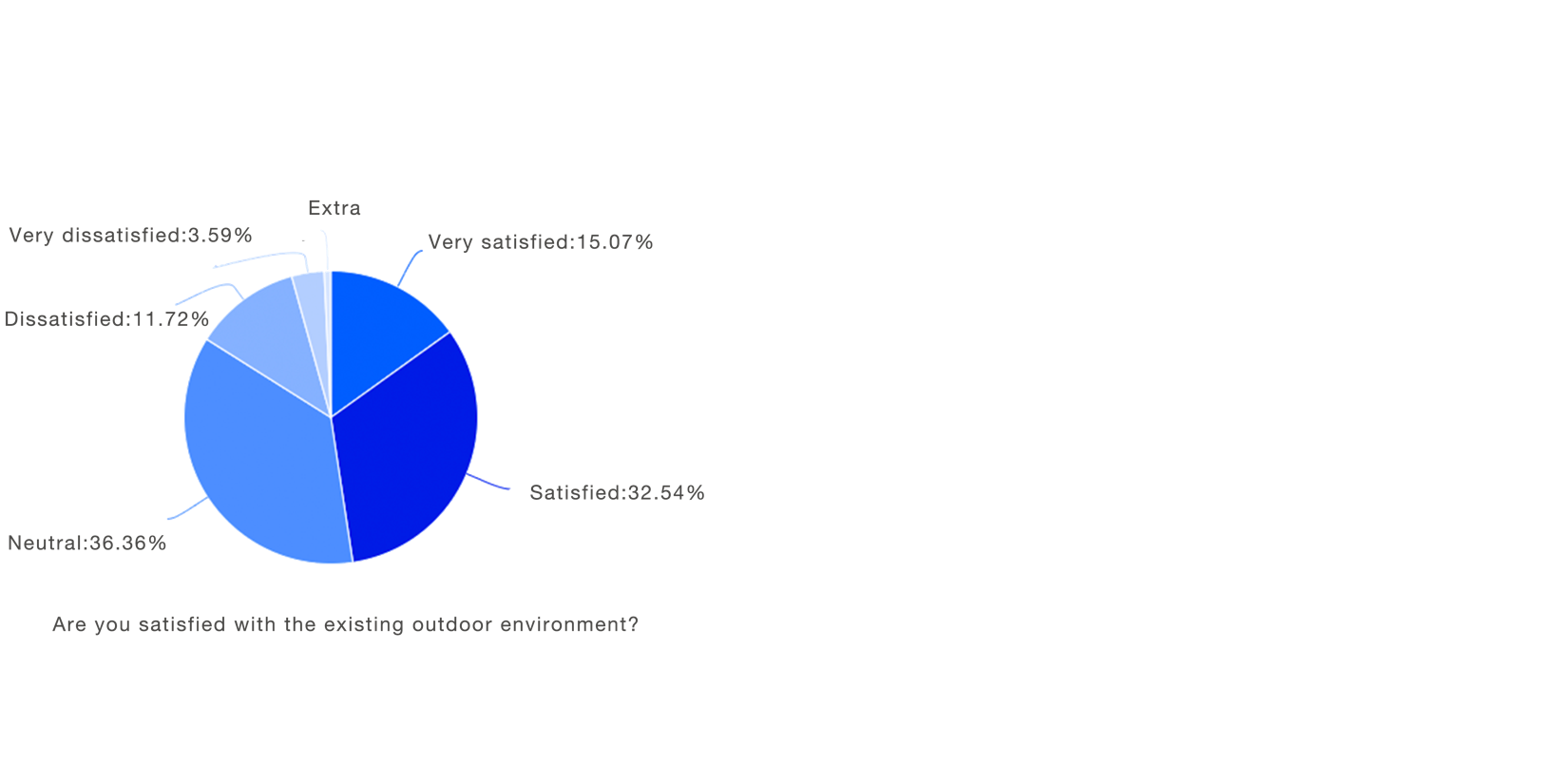


4. At what time of the day do you usually use the outdoor environment? [Single choice question]


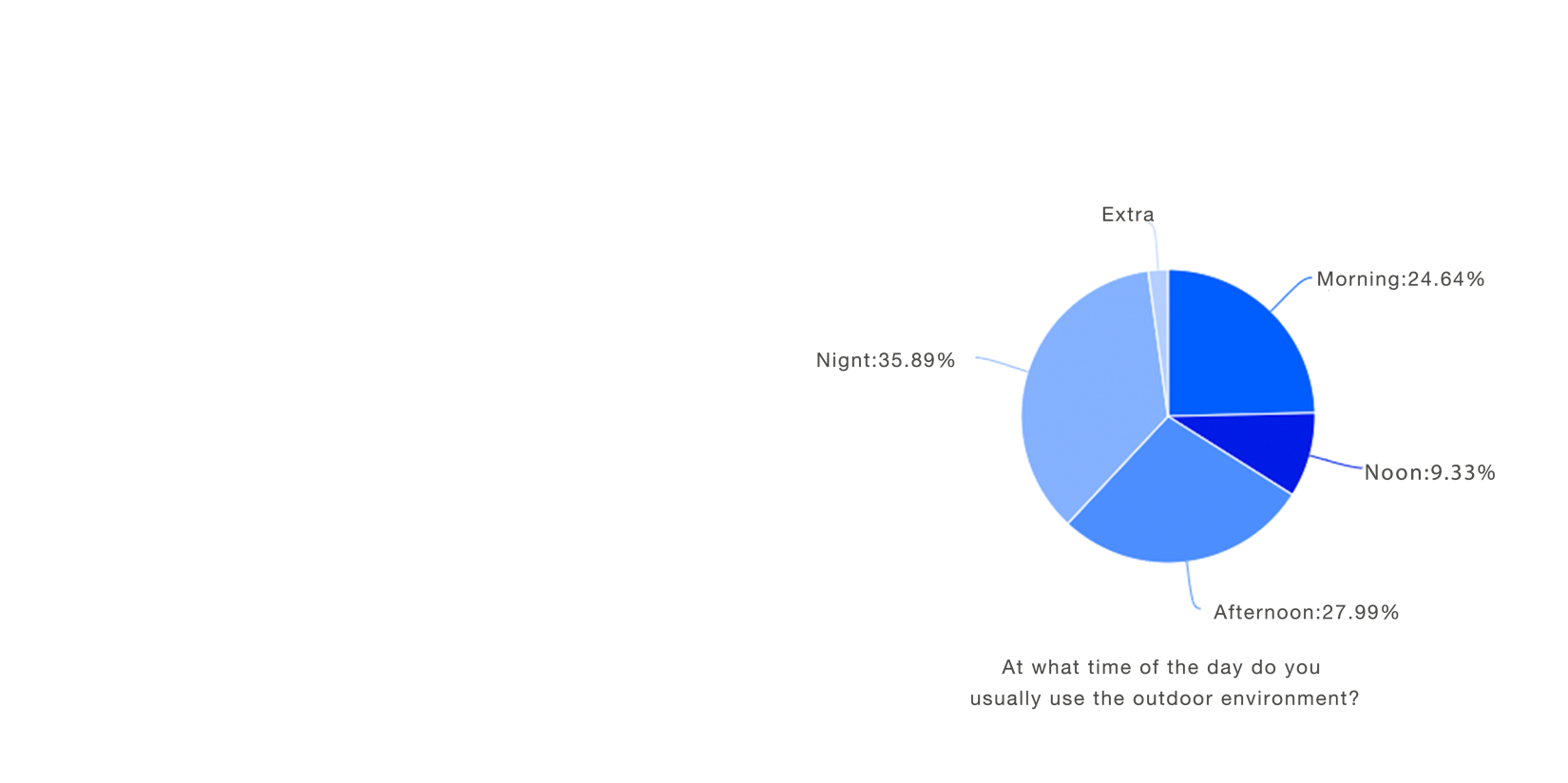


5. How important is it for you to have a garden outside Zhongnan Hospital? [Single choice question]


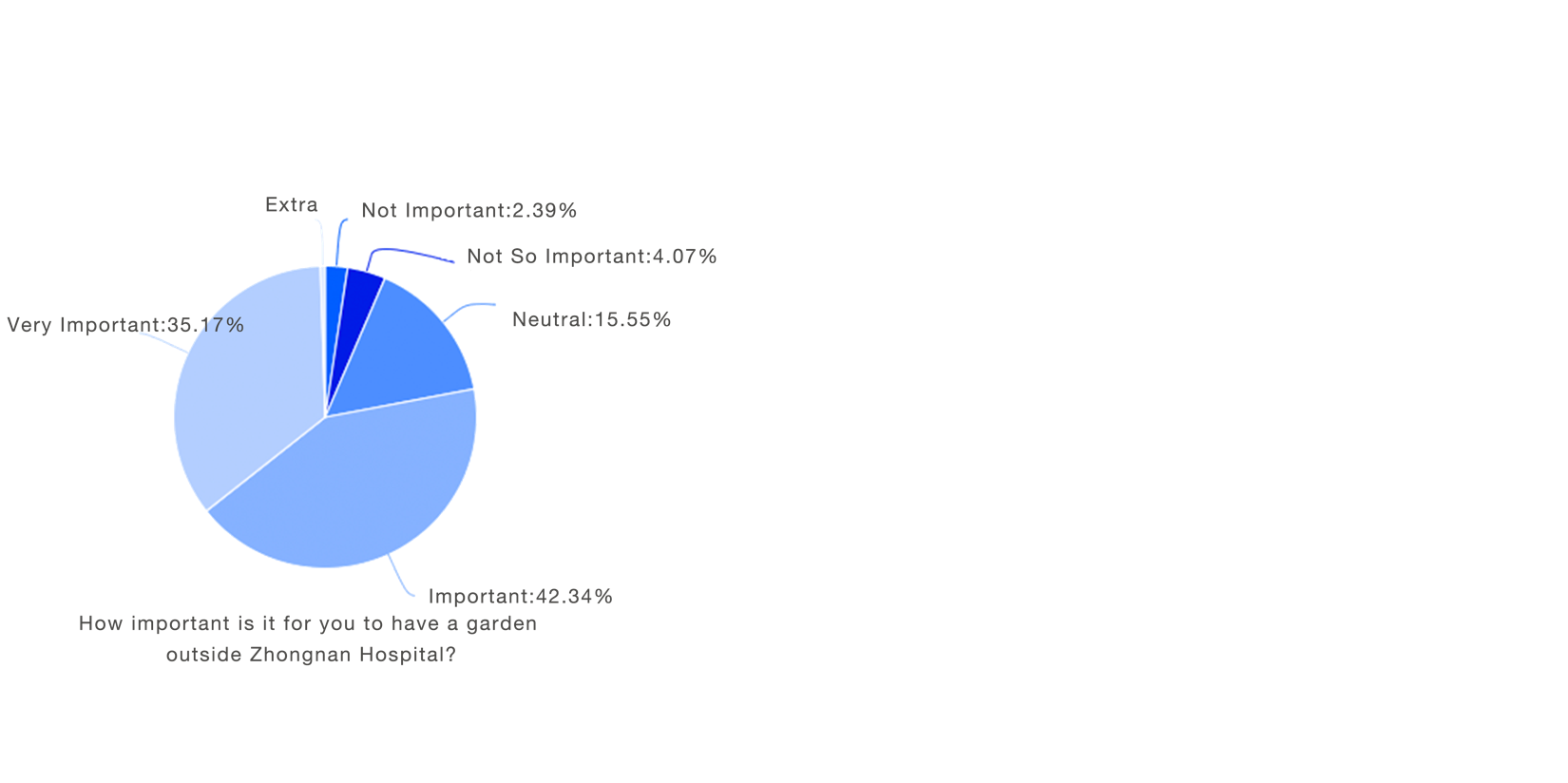


6. What do you generally do in the existing outdoor environment? How long does it take?


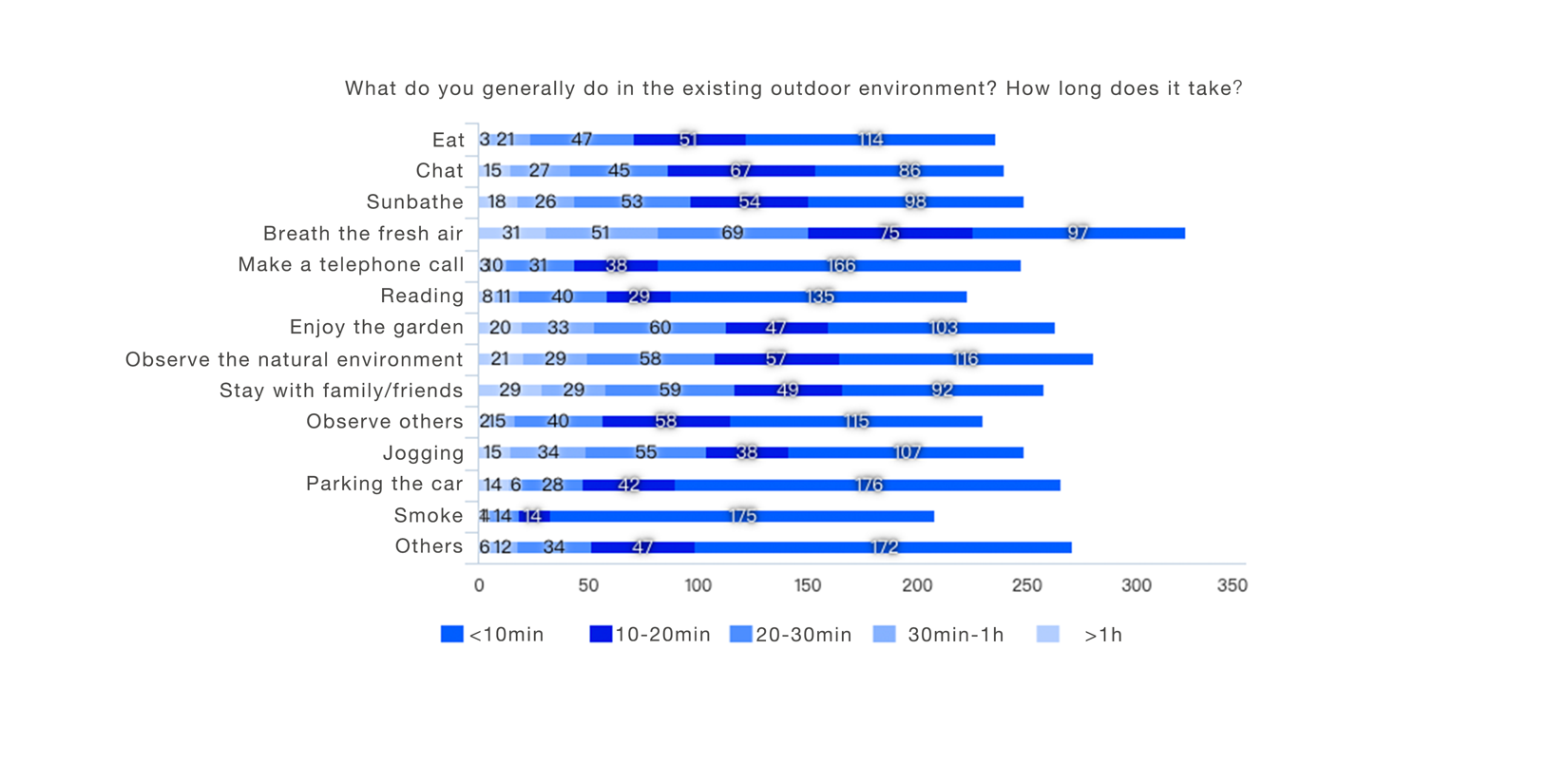


7. Do you want some rehabilitation projects to be carried out outdoors？[Single choice question]


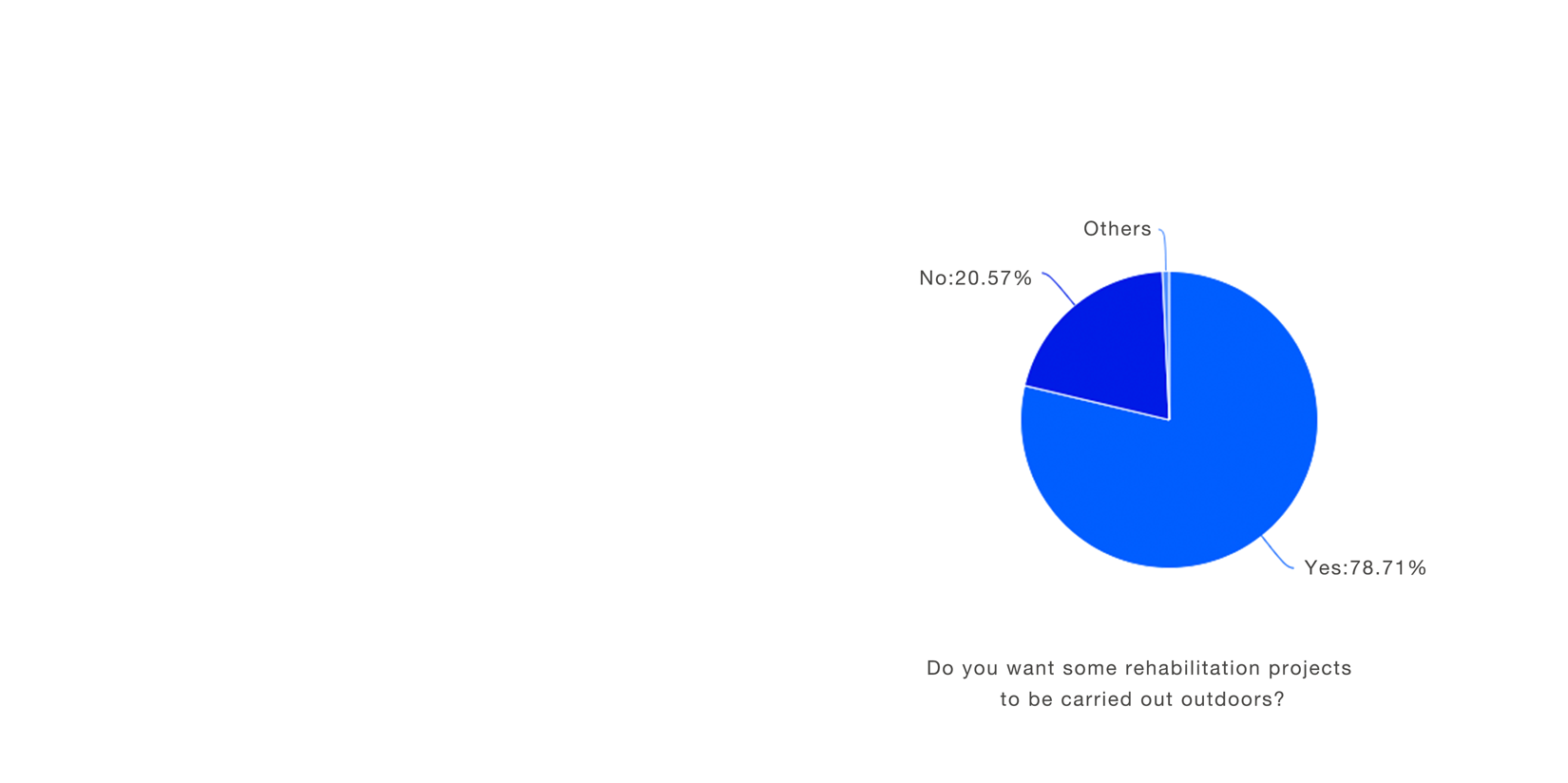


8. From 1 to 5, How do you rate these aspects of the outdoor environment?


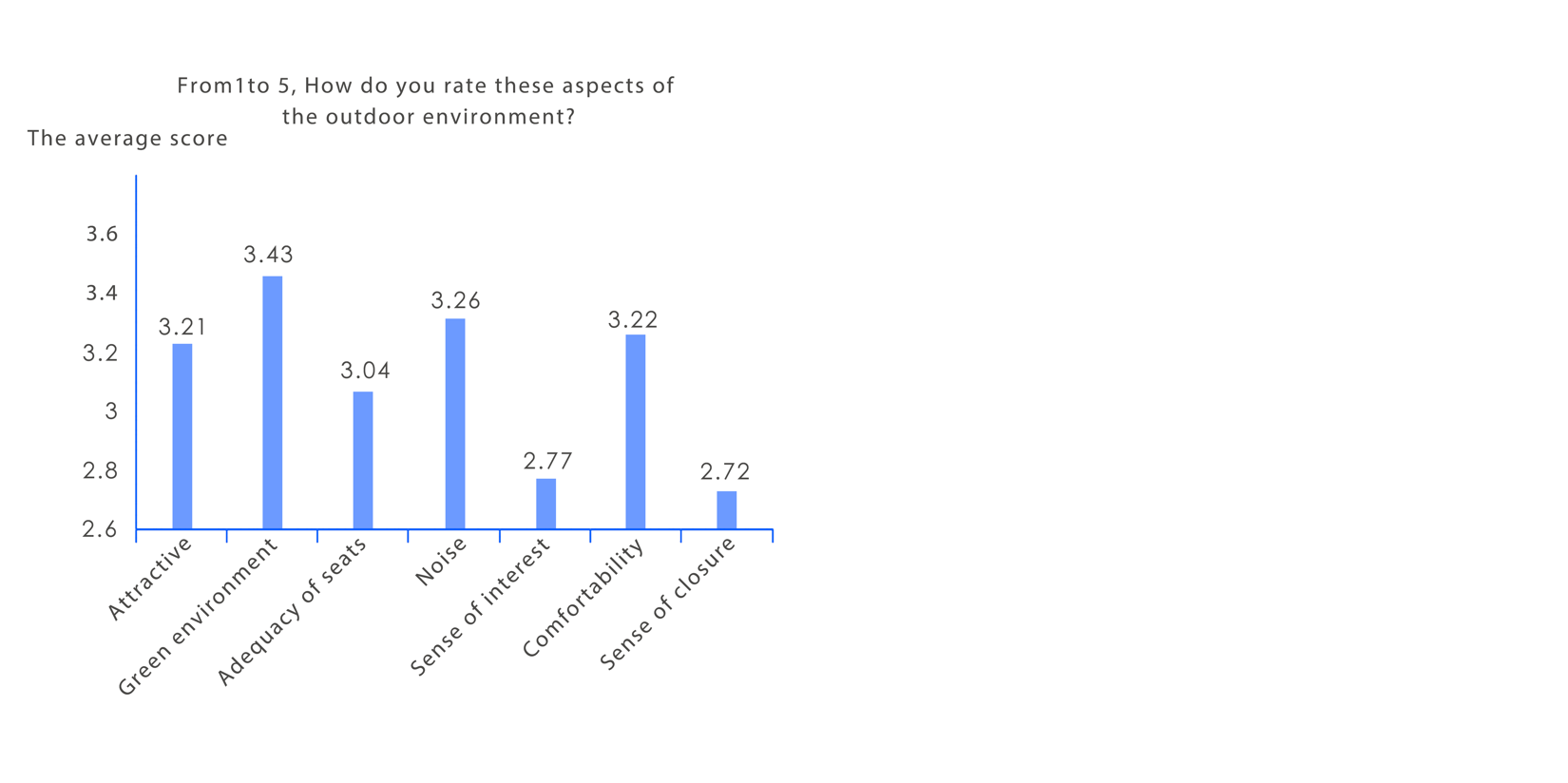


9. Do you want a place to sit？ [Single choice question]


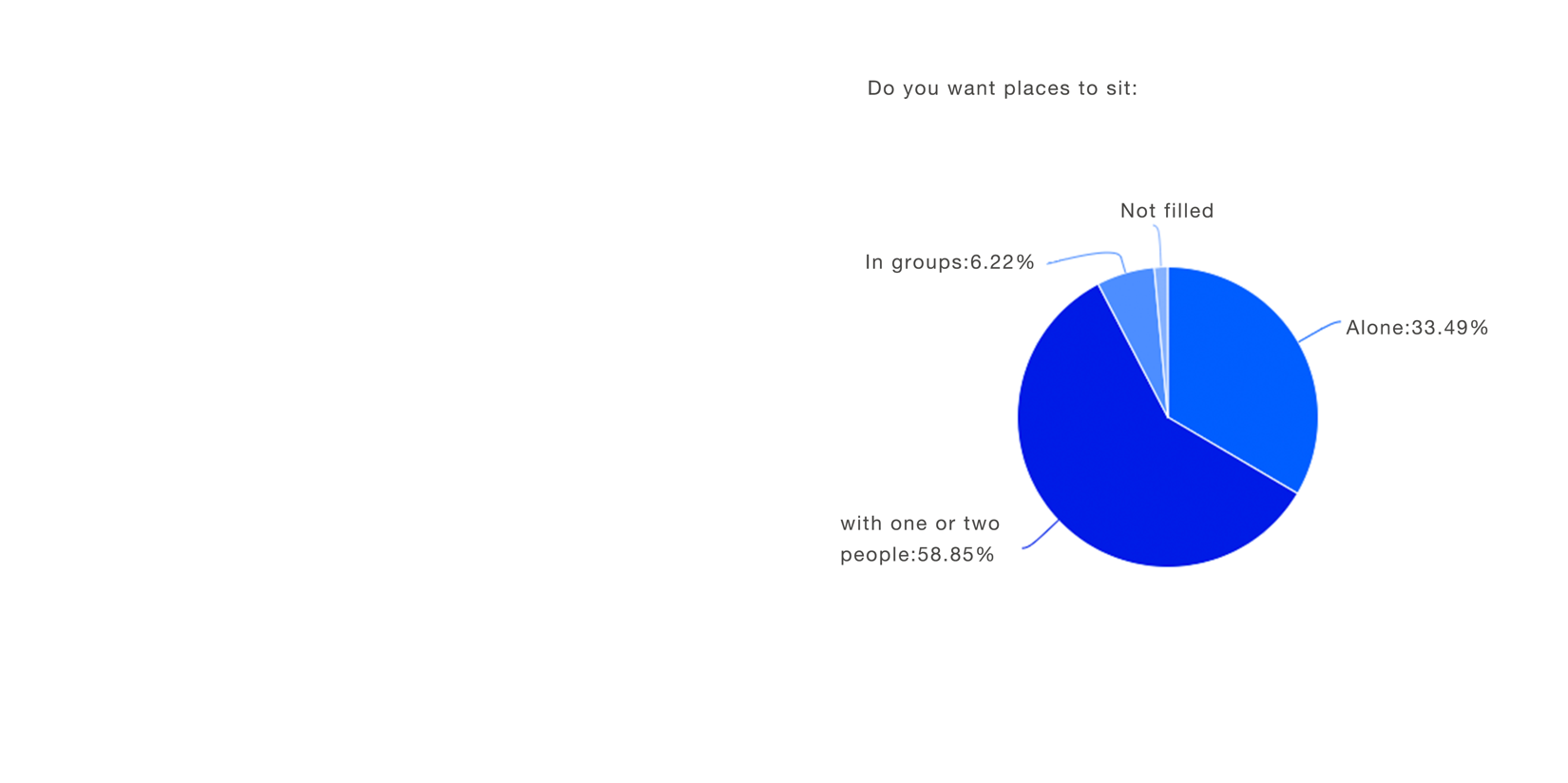


10.If you are hospital staff in Zhongnan Hospital, do you need a dedicated outdoor garden for you and your colleagues? [Single choice question]


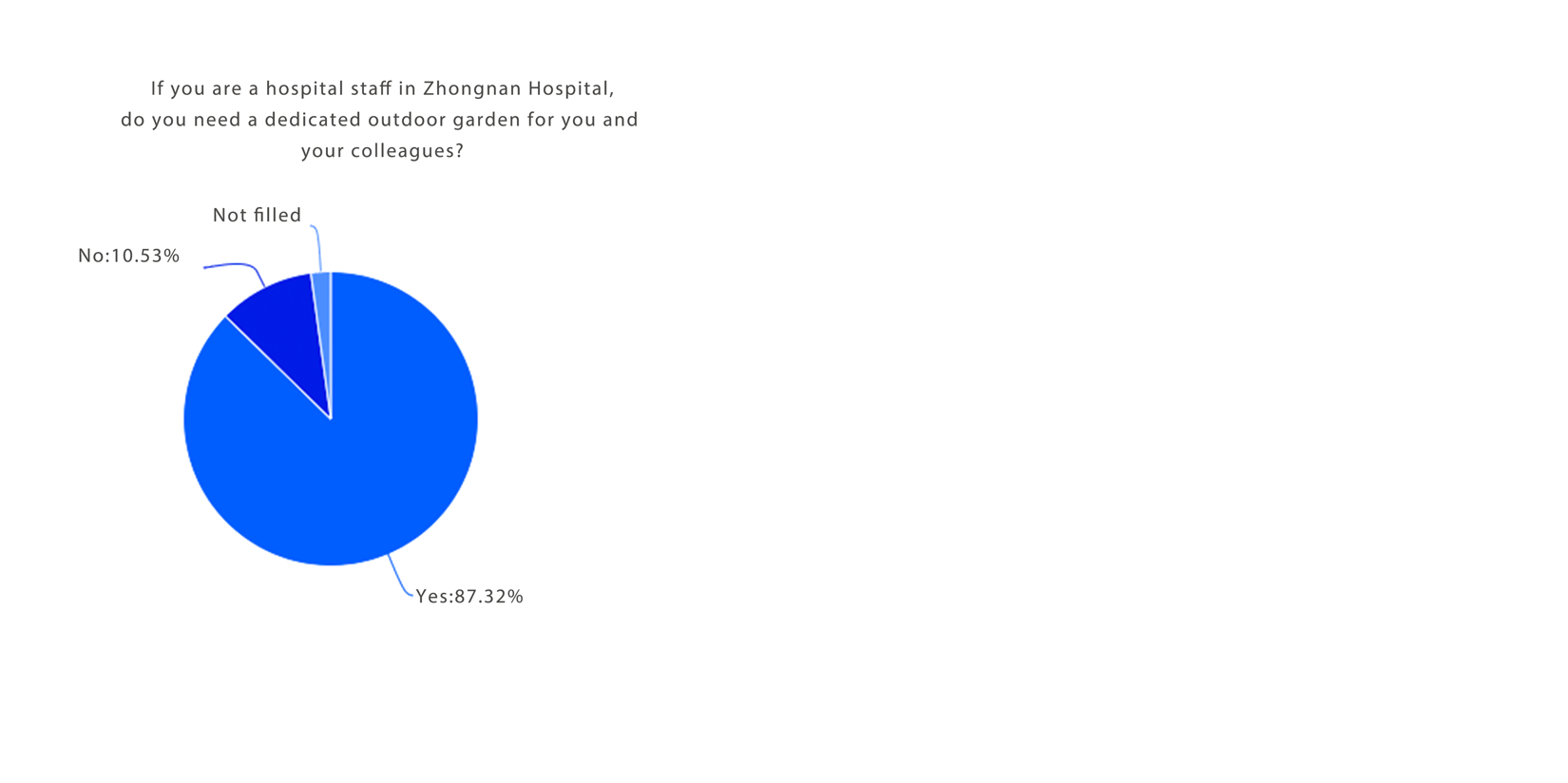


11. What features do you expect in the existing outdoor environment in the Zhongnan Hospital? [Multiple choice questions]


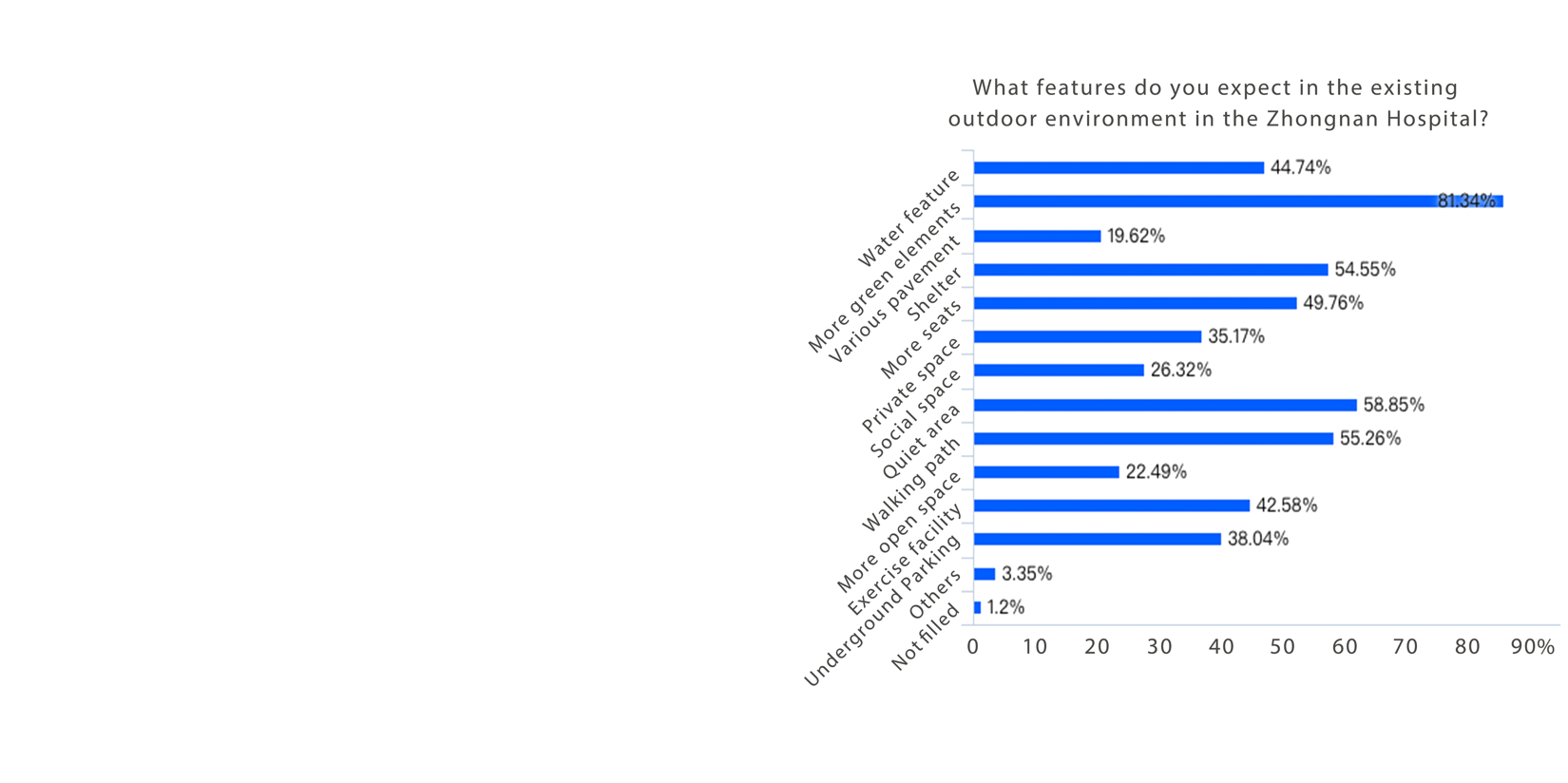


12. If you are a healthcare provider, what is the stress index of your relationship with patients? (The pressure increases from 1 to 5) [Single choice question]


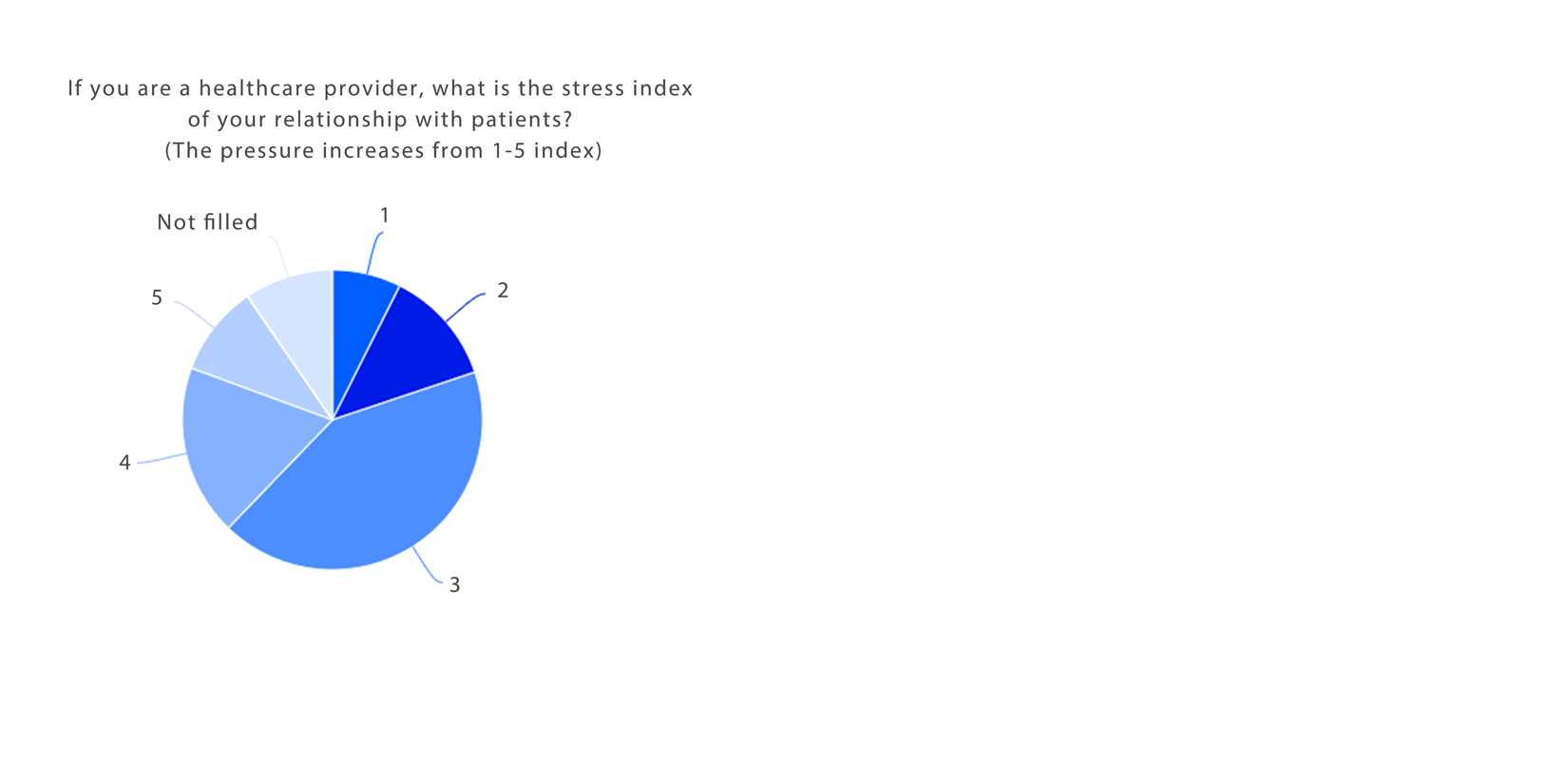


**Appendix 3: Questionnaire results （open question answer）**

13. What are your satisfactions and dissatisfactions with the existing outdoor environment of Zhongnan Hospital?

| NO | | Answer |
| --- | --- | --- |
| 3 | Too many cars, too many people | |
| 6 | There are too many cars, it is very unfriendly to pedestrians, and the outdoor environment is not well utilized! Basically a parking lot. | |
| 10 | Get rid of the stinky and notorious red building behind! | |
| 11 | None | |
| 12 | Too dilapidated, the slope is too steep, and it is connected with the school. | |
| 13 | There are too many cars, which affects the traffic and is not safe, and also affects the air quality. It is better to introduce the parking lot underground | |
| 14 | I hope there are many green plants and convenient and safe pedestrian passages. | |
| 15 | The odor of garbage removal and disposal in the north affects the playground | |
| 17 | The medical environment is quite satisfactory! Parking is not satisfactory. ' | |
| 18 | Still needs improvement, I didn't even notice the greenery | |
| 19 | No underground parking lot, small activity space. | |
| 20 | Too few parking spaces | |
| 23 | It's too cold, and it doesn't belong to the environment of a high-end high-level hospital at all. | |
| 28 | Parking is too difficult! | |
| 29 | Zhongnan Hospital is located on Donghu Road, which is a part of Wuhan Avenue. There are key landmarks such as Shuanghu Bridge, Chuhe Han Street, Hanxiu, Provincial Museum, and Provincial Government next to it. The external environment and conditions are very good. If there is a strong underground parking lot, it would be even better to change the current surface parking lot into a green space, a corridor, a garden... | |
| 30 | Lack of greenery and public seats, too few shops | |
| 31 | Small space | |
| 32 | Too many cars | |
| 33 | Dissatisfied: The traffic lanes are congested, traffic jams often occur in the courtyard, and the road surface is uneven and rainy. Satisfied: The overall layout looks tidy | |
| 36 | Dirty and messy | |
| 37 | Dissatisfied | |
| 38 | Medical waste affects the living environment | |
| 40 | Life is inconvenient! | |
| 41 | Too many cars, too little greenery | |
| 42 | The dump is too close to the hospital and residential buildings | |
| 43 | Crowded | |
| 46 | It's too crowded, there's nowhere to even walk | |
| 49 | The proportion of greenery is low, and there are few parking spaces. | |
| 50 | Cars occupy an area that is inconvenient for transportation, affecting the commute to and from work | |
| 51 | There are basically no pedestrian passages, and there is basically no reasonable planning in the hospital, which is extremely chaotic | |
| 56 | None | |
| 59 | None | |
| 60 | More shade | |
| 61 | Wish there was more space and fun | |
| 63 | None | |
| 65 | Dissatisfied with the loud outdoor noise, and the entry and exit of vehicles cause great trouble to pedestrians. | |
| 69 | Fine | |
| 79 | Five | |
| 82 | Hope it's more interesting | |
| 92 | Satisfaction point: It is near the lake, and the scenery outside the hospital is good. Dissatisfaction point: There are few parking spaces during peak hours, no garden, and no shady trees on the road to the hospital building. | |
| 93 | None | |
| 95 | . | |
| 99 | None | |
| 100 | Too many vehicles | |
| 101 | There are no leisure places, and the patients can only be on the playground of the medical department, which somewhat affects the activities of the students | |
| 107 | I wish there were more plants | |
| 113 | No | |
| 117 | Hope to be greener | |
| 119 | None | |
| 127 | I personally think that the green environment is a very necessary place for doctors and patients, and it helps to relax and relieve stress. | |
| 129 | No | |
| 132 | Satisfied | |
| 135 | None | |
| 140 | Basically satisfied | |
| 141 | Don't feel anything | |
| 143 | None | |
| 146 | Fine | |
| 148 | The playground behind Zhongnan Hospital is used by patients and students at the same time, and affect each other | |
| 149 | Bad air, cars everywhere | |
| 150 | Not enough greenery | |
| 151 | None | |
| 153 | Uneven ground | |
| 155 | There is none left | |
| 156 | It's pretty good, it's not bad to come to extract teeth, it's very fast | |
| 157 | None | |
| 158 | None | |
| 159 | None | |
| 162 | None | |
| 167 | Can provide more seats | |
| 171 | and | |
| 173 | No | |
| 175 | The garbage can in the site isn’t not always clean, and there is no place to rest in the playground | |
| 178 | The environment is beautiful and pleasant | |
| 184 | Now the attitude of the security guard is much better, basically satisfied | |
| 187 | Too many cars | |
| 188 | The back of Building 4 is very noisy at night or early in the morning. I don't know if it is the physical environment or the garbage dump, which affects the rest of the staff on duty. | |
| 189 | The road from the outpatient department of our hospital to Building 6/7 is narrow and bumpy. There is no segregation between pedestrians, vehicles, and hospital beds. No widening. The site didn’t change too much and still has many problems | |
| 190 | There are many vehicles, it is recommended to separate the roads for vehicles and people. the road is not good | |
| 192 | The planning of the hospital is more concentrated and more open | |
| 193 | None | |
| 194 | Too many people, noisy roads, few green plants, few seats | |
| 195 | The outdoor space of Zhongnan Hospital is still very large and cheerful, but it is basically used as a parking lot, which is noisy. It is extravagant to build a garden in this place where every inch of land is expensive, but reasonable planning can yield unexpected gains | |
| 199 | None | |
| 200 | None | |
| 203 | Too many cars, too few flowers | |
| 204 | Difficult parking | |
| 207 | The road is uneven and there is no subway | |
| 212 | The parking lot in front of Building No. 6 is full of stones. It is dusty on sunny days and waterlogged on rainy days. The location of the large trash can is too obvious, making it feel untidy | |
| 214 | People and vehicles are not separated, the road is congested | |
| 215 | The gate of the hospital is narrow, and there is only one lane for the entrance and exit on both sides of the gate. The road surface of the whole hospital is uneven, and the greenery basically has no sense of matching and design. There is no shade at all for parking in the big square, only the reflection of the roof. | |
| 216 | Too little shade | |
| 219 | Few places to eat | |
| 220 | I hope the road will be flatter, the transfer of patients will not be bumpy, and there will be more parking spaces. | |
| 223 | Repair the road. It’s really hard work to turn a patient around. I’m so tired and sweaty, and the distance between buildings is still far away. I really need the road to be repaired. | |
| 224 | There are too many vehicles, it is difficult to take patients for examination every time | |
| 225 | Chaotic parking, narrow roads, unreasonable arrangement of road railings | |
| 228 | There is no channel dedicated to transferring patients between buildings, which is too bumpy. | |
| 229 | Normal | |
| 231 | The road is too potholed, too many cars parked, too messy and crowded | |
| 232 | Too noisy, the road is potholed | |
| 233 | None | |
| 234 | Too few seats, the road is too narrow, traffic jams | |
| 235 | Potholes in the road | |
| 236 | Can there be a coffee shop | |
| 237 | Insufficient greenery | |
| 240 | It's too congested, it's very inconvenient to push the bed | |
| 243 | Just a big parking lot | |
| 244 | Strengthen greening | |
| 245 | There are too few places to buy things, which can't meet the needs of patients and medical staff. Especially in terms of diet, there is no way to provide a variety of diets. There are no small shops, which may be related to the geographical location and are relatively close. | |
| 246 | Fine | |
| 247 | Too messy | |
| 250 | It is very inconvenient to do CT for critically ill patients, the road is uneven, and the transfer is risky | |
| 253 | There are many cars, | |
| 255 | There is no dedicated patient transfer road, and the existing road surface is too poor, and pedestrians and motor vehicles must be avoided. Motor vehicles often drive by quickly and almost hit medical staff and their families. | |
| 257 | Lanes and Humanities Intertwine | |
| 258 | The electric car canopy is missing | |
| 261 | None | |
| 262 | Satisfied with the greening; relatively unsatisfied with the entertainment facilities | |
| 263 | The road surface is uneven. Pedestrians, vehicles, hospital beds, etc. are fighting each other on the crowded road. At least there should be a special channel for transferring patients. When it is windy and rainy, patients would not have to lie on the hospital trolley-bed in the rain | |
| 264 | There are too many cars, and there is no dedicated channel for transferring patients. | |
| 265 | The vehicles in the morning peak and evening peak are too crowded, and there are too few parking spaces for employees | |
| 266 | Parking is troublesome and messy | |
| 267 | Too much dust, narrow sidewalks, badly damaged pavement. | |
| 268 | It is a waste of such a large space to use as an above-ground parking lot. It should be made into several floors underground, with sufficient parking spaces, which saves time in and out of the hospital every day, does not cause traffic jams, and makes pedestrians on the ground safer. The space on the ground can be reused. Perfect. | |
| 269 | None | |
| 271 | All ok | |
| 272 | Few parking spaces, poor road conditions | |
| 273 | Few parking spaces | |
| 275 | 1. Too much noise. 2. The distance between the buildings is not smooth. When transferring patients, the patients often complain of discomfort or even vomit! Therefore, there are great opinions on the transfer of medical care. 3. Parking is difficult. There was a patient who was transferred to another department and contacted his family members. The family members spent an hour looking for a parking space downstairs. 4. | |
| 276 | Building No. 6 needs a service elevator to transport garbage, medicine, and patients. It is too dirty and irregular. It’s dark at night, and there are no streetlights. Every time I transfer a patient or send a patient for an examination, the road is uneven and there are too many potholes. I often transfer patients in the rain. There is no dedicated rain sheltered corridor to transfer patients. I was having my period, and I have to transport the patient in the rain, the patient is even more uncomfortable. | |
| 279 | It is too inconvenient to park electric vehicles, people and vehicles are not separated, messy! | |
| 280 | There are too many cars on the road, it is messy, there is no passage for transferring patients, and there is no place suitable for patients and employees to walk and exercise | |
| 282 | None | |
| 283 | None | |
| 285 | No yet | |
| 286 | No | |
| 289 | Too little outdoor greenery | |
| 293 | None | |
| 295 | The function of each department is not clear | |
| 296 | Smoking area for patients and dedicated garden for medical staff | |
| 301 | Too little greenery, too few parking spaces, narrow roads | |
| 304 | Now I feel that there is no outdoor environment, and I can have a place to rest, walk, and talk | |
| 306 | Need more greenery, flowers | |
| 310 | None | |
| 311 | Parking spaces everywhere | |
| 314 | None | |
| 315 | I hope there is a place for outdoor rest and relaxation, which can relieve stress | |
| 316 | None | |
| 317 | Parking management is poor, random parking is not according to the parking space, frequent congestion, basically there is no outdoor environment | |
| 318 | Not enough exercise facilities | |
| 320 | Normal | |
| 321 | None | |
| 322 | Uneven road | |
| 324 | None | |
| 325 | Parking location | |
| 326 | None | |
| 330 | None | |
| 331 | None | |
| 333 | None | |
| 334 | Good | |
| 338 | Too many cars | |
| 340 | Parking is everywhere on the ground, it is unsafe and inconvenient to transfer patients, and the environment is too messy, which lowers the grade of the whole hospital | |
| 344 | Too little greenery | |
| 346 | There are too few parking spaces, the hospital square is full of parked cars, and there is no external environment | |
| 347 | None | |
| 348 | It is too inconvenient to transfer on a flat car or cart. It is impossible to transfer when it rains, and it is not easy to push a wheelchair. | |
| 350 | I am more satisfied with the large space, but less satisfied with the fact that cars and pedestrians share the same road, and it is easy to get stuck in traffic | |
| 351 | There are too few parking spaces, serious traffic jams, and the parking fees for employees are too expensive | |
| 352 | None | |
| 353 | Not enough parking spaces | |
| 356 | Reduce ground parking spaces and provide more passages for pedestrians alone | |
| 358 | Not enough outdoor greenery | |
| 359 | Few parking spaces, difficult to park | |
| 362 | Too noisy too many cars. | |
| 365 | None | |
| 366 | None | |
| 367 | Satisfied | |
| 368 | Not very good looking | |
| 370 | There are cars everywhere, and the places to walk are too limited. I am satisfied that the surrounding area is neatly planned, there are no small stalls and hawkers, and the lakes and mountains are beautiful. | |
| 374 | Parking takes up too much personal space | |
| 376 | The location of the hospital is good. Dissatisfaction is human. The car is mixed. There are too few green plants in front of Building 4. There are too few parking spaces. If you want to dig an underground parking lot, you need three floors. | |
| 379 | Dissatisfied: Too crowded, no parking space, not enough green plants | |
| 383 | The road to building 6 is uneven | |
| 385 | There is a parking lot directly in front of Building 4. Parking will be convenient and direct, but since the pine trees were cut down, there are only parking lots, a few sparse trees, and construction sites outside, which is very messy. Now the environment of the hospital is dependent on the Fruit Lake and East Lake. It is hoped that the areas within the hospital and outside the building can be better planned and utilized, so that our hospital can be further improved! | |
| 387 | None | |
| 390 | Miscellaneous, difficult, poor, crowded. | |
| 392 | There are cars outside | |
| 393 | Our hospital doesn't have any outdoor environment, it's all borrowed sand lakes, and they are all built by the government | |
| 395 | Messy | |
| 397 | None | |
| 399 | Dissatisfaction: There are too few places to sit | |
| 400 | No | |
| 401 | None | |
| 404 | None | |
| 406 | Dilapidated and backward | |
| 407 | None | |
| 409 | Staff canteens should be separated from patient canteens to avoid cross-infection. The environment in the residential area is dirty and messy. | |
| 410 | Piles of medical waste in the basement of 20 buildings in the medical waste and family area, which poses a major safety hazard | |
| 411 | There are too few parking spaces, and the family area is not isolated from patients. | |
| 412 | Dirty and messy, the family area is not separated from the medical area, patients are running around everywhere, it is inconvenient for patients to buy and eat | |
| 415 | Unclean | |
| 416 | Junkyard exposed | |
| 418 | Why does the open space outside the west gate of Building 20 become a dump for domestic garbage, even hospital decoration materials and home decoration materials? Every time it needs a forklift to clear it, the dust raised makes people feel that it is no different from a garbage dump. | |
| 419 | There should be an overall plan to create an elegant, quiet, green, and civilized environment | |

Blank answers have been filtered out.
